# Histone H3K9 and H4 Acetylations and Transcription Facilitate the Initial CENP-A^HCP−3^ Deposition and De Novo Centromere Establishment in *Caenorhabditis elegans* Artificial Chromosomes

**DOI:** 10.1186/s13072-018-0185-1

**Published:** 2018-04-13

**Authors:** Jing Zhu, Kevin Chi Lok Cheng, Karen Wing Yee Yuen

**Affiliations:** School of Biological Sciences, The University of Hong Kong, Kadoorie Biological Sciences Building, Pokfulam Road, Pokfulam, Hong Kong

**Keywords:** De novo centromeres, Neocentromeres, Centromere establishment, Artificial chromosomes, Epigenetics, Chromatin environment, Histone modifications, Histone acetylations

## Abstract

**Background:**

The centromere is the specialized chromatin region that directs chromosome segregation. The kinetochore assembles on the centromere, attaching chromosomes to microtubules in mitosis. The centromere position is usually maintained through cell cycles and generations. However, new centromeres, known as neocentromeres, can occasionally form on ectopic regions when the original centromere is inactivated or lost due to chromosomal rearrangements. Centromere repositioning can occur during evolution. Moreover, de novo centromeres can form on exogenously transformed DNA in human cells at a low frequency, which then segregates faithfully as human artificial chromosomes (HACs). How centromeres are maintained, inactivated and activated is unclear. A conserved histone H3 variant, CENP-A, epigenetically marks functional centromeres, interspersing with H3. Several histone modifications enriched at centromeres are required for centromere function, but their role in new centromere formation is less clear. Studying the mechanism of new centromere formation has been challenging because these events are difficult to detect immediately, requiring weeks for HAC selection.

**Results:**

DNA injected into the *Caenorhabditis elegans* gonad can concatemerize to form artificial chromosomes (ACs) in embryos, which first undergo passive inheritance, but soon autonomously segregate within a few cell cycles, more rapidly and frequently than HACs. Using this in vivo model, we injected LacO repeats DNA, visualized ACs by expressing GFP::LacI, and monitored equal AC segregation in real time, which represents functional centromere formation. Histone H3K9 and H4 acetylations are enriched on new ACs when compared to endogenous chromosomes. By fusing histone deacetylase HDA-1 to GFP::LacI, we tethered HDA-1 to ACs specifically, reducing AC histone acetylations, reducing AC equal segregation frequency, and reducing initial kinetochroe protein CENP-A^HCP−3^ and NDC-80 deposition, indicating that histone acetylations facilitate efficient centromere establishment. Similarly, inhibition of RNA polymerase II-mediated transcription also delays initial CENP-A^HCP-3^ loading.

**Conclusions:**

Acetylated histones on chromatin and transcription can create an open chromatin environment, enhancing nucleosome disassembly and assembly, and potentially contribute to centromere establishment. Alternatively, acetylation of soluble H4 may stimulate the initial deposition of CENP-A^HCP−3^-H4 nucleosomes. Our findings shed light on the mechanism of de novo centromere activation.

**Electronic supplementary material:**

The online version of this article (10.1186/s13072-018-0185-1) contains supplementary material, which is available to authorized users.

## Background

The centromere is a chromatin region specialized for chromosomes segregation. Its function is to attach the chromosome through the kinetochore complex to the microtubule spindle fibers, which pull sister chromatids apart during anaphase in mitosis. Centromeric regions have different DNA sequences and sizes across species, but functional centromeres are often marked by the conserved histone H3 variant, CENP-A. Neocentromeres occasionally form on ectopic regions after chromosomal rearrangements or inactivation of the original centromere [1]. Centromere repositioning also occurs during speciation despite gene synteny maintenance [2]. Moreover, de novo centromeres can form on exogenous DNA transformed into cells, which then segregates faithfully as artificial chromosomes (ACs). These functional neocentromeres and de novo centromeres also contain CENP-A. Thus, CENP-A is believed to be the primary epigenetic mark to maintain centromere identity. CENP-A is also recognized as one of the most upstream proteins in the kinetochore assembly hierarchy and acts as a platform to recruit other kinetochore proteins [3, 4].

Interestingly, CENP-A nucleosomes replaces some canonical H3 nucleosomes in the centrochromatin region [5], but CENP-A nucleosomes do not occupy all centrochromatin in regional monocentromeres and holocentromeres studied. Instead, they intersperse with H3 nucleosomes [6, 7]. Specific histone modifications on H3 and CENP-A nucleosomes are enriched at centromeres, and some are important for centromere function. For example, histone H3 lysine 4 dimethylation (H3K4me2), which is associated with active transcription, is enriched at human and *Drosophila* centromeres by chromatin fiber analysis, while H3 and H4 acetylations and heterochromatin marks, H3K9me2/3, are under-represented in the centrochromatin region [8–10]. This shows that the centrochromatin resembles neither typical euchromatin nor heterochromatin. H3 and H4 hypoacetylations are important for centromere function in *S. pombe* [11]. On the contrary, an enrichment of H3K9me3 and a low level of H3K4me2 are found in centromeres of chicken DT40 cells [12]. H4K20me1, which is associated with transcriptional activation [13], is highly enriched in human and chicken CENP-A nucleosomes, and is required for kinetochore function [14]. H3T3ph, which is associated with mitosis, is also found in human and chicken centromeres and is responsible for the localization of Aurora B [12, 15, 16]. H2B monoubiquitination (H2Bub1), which is involved in replication, transcription and DNA repair, is found in human and *S. pombe* centromeres and is important for proper chromosome segregation in *S. pombe* and *S. cerevisiae* [17–22]. In holocentric *C. elegans*, H3K27me3 and H3K9me3 positively correlate with CENP-A domains [7, 23, 24], but a reduction in H3K27me3 did not affect CENP-A localization [25]. In addition, several CENP-A modifications have been shown to be important for centromere function, including human CENP-A glycine 1 (G1) trimethylation, which aids the interaction with alpha-satellite DNA; S16 and 18 phosphorylation, which compacts the CENP-A nucleosomal array [26]; and K124 ubiquitination, which is required not only for the maintenance of the old CENP-A, but also for the recruitment of the newly synthesized CENP-A [27]. However, whether these histone modifications only regulate existing centromere maintenance, or also affect centromere activation and inactivation are not clear.

The processes of centromere activation and inactivation, despite important for chromosome stability, are not easy to capture and study due to their infrequent occurrences. For instance, human ACs (HACs) form at a low frequency (0–30%) after the introduction of naked alpha-satellite repetitive DNA and require 6 weeks of selection [28]. Recently, the development of HACs carrying a synthetic, higher-order alpha-satellite repeat array with tet operator (tetO) sequence inserted in every other alphoid monomer has facilitated the modulation of the de novo centromere chromatin environment on HACs by targeting the tet repressor (tetR) fused with a specific histone modifier or transcriptional regulator [29, 30]. The effects of several histone modifications have been studied on stably maintained de novo centromeres on HACs, which have already propagated through multiple cell cycles. These stably maintained de novo centromeres, as in natural centromeres, contain pre-existing CENP-A nucleosomes to build the kinetochore in mitosis and mark the centromere identity for propagation through cell cycles and generations. H3K4me2 was shown to be required for CENP-A chaperone HJURP targeting and CENP-A assembly on stably maintained de novo centromeres on HACs [31]. However, tethering of either a transcriptional activator or repressor induces different degrees of HAC loss, suggesting that a balance between open and closed chromatin and transcriptional level may be required for the maintenance of de novo centromeres [29, 30]. The propagating mechanisms of de novo centromeres potentially resemble those of endogenous centromeres.

On the other hand, during the de novo centromere formation and stabilization process, new CENP-A nucleosomes are first incorporated at an ectopic site and subsequently propagated at this specific location. New centromere establishment may require different or additional histone modifications when compared with centromere propagation. However, the role of histone modifications in de novo centromere formation or maturation is not well understood. A previous study in HACs showed that H3K9ac and H3K9me3 promotes and inhibits HAC formation, respectively [32]. Despite the recent advances in HAC construction methods [33], generation of a large number of HACs to directly observe de novo centromere formation events remains difficult due to the low HAC formation rate and long selection time. In addition, HACs could only be formed in a limited number of human cell lines, possibly due to the differences in the chromatin environment [28, 32]. This may limit the use of HACs as de novo centromere models and their applications as cloning vectors.

In order to study the process of de novo centromere formation more directly, an in vivo system for rapidly generating visualizable new ACs in *Caenorhabditis elegans* has been developed [34]. In *C. elegans*, DNA injected into the gonad can concatemerize to form ACs, also known as extrachromosomal arrays (Ex), in embryos produced from the injected gonad [35, 36]. These ACs are commonly used to express transgenes in worms. Yet, even non-*C. elegans* DNA sequences are capable of forming ACs, possibly because the regulation of centromeres depends heavily on epigenetics and not on DNA sequences in holocentromeres [7]. By live-cell imaging, these newly formed, first-generation ACs first undergo passive inheritance, but soon gain autonomous segregation ability within a few cell cycles [34]. By immunofluorescence, inner and outer kinetochore proteins assemble on ACs that have been propagated for a few generations, indicating the establishment of de novo centromeres [34]. Interestingly, these ACs do not hitch-hike on endogenous chromosomes, but congress and align independently onto the metaphase plate [34]. Importantly, *C. elegans* ACs gain de novo centromeres more rapidly (by 64-cell stage, after 6 cell divisions, within 200 min of fertilization) [37] and at a higher frequency than HACs. Previously, we showed that inhibition of the heterochromatin protein HP1 increases the segregation frequency in 1-cell stage, suggesting heterochromatin may hinder de novo centromere formation [34]. Here, we utilize this system to study the role of histone modifications and transcription in *C. elegans* ACs. We show that newly formed *C. elegans* ACs are hyperacetylated at H3K9 and H4K5/8/12/16 compared to endogenous chromosomes, and this hyperacetylation and associated transcription promote efficient de novo centromere formation on *C. elegans* ACs. Our work demonstrates the use of *C. elegans* ACs as an in vivo model to study the role of epigenetic factors in de novo centromere formation during the actual time of the event, and illustrates the involvement of histone acetylation and transcription in de novo centromere establishment.

## Results

### *Caenorhabditis elegans* newly formed ACs in early cell stage are hyperacetylated at H3K9 and H4

**Fig. 1:**
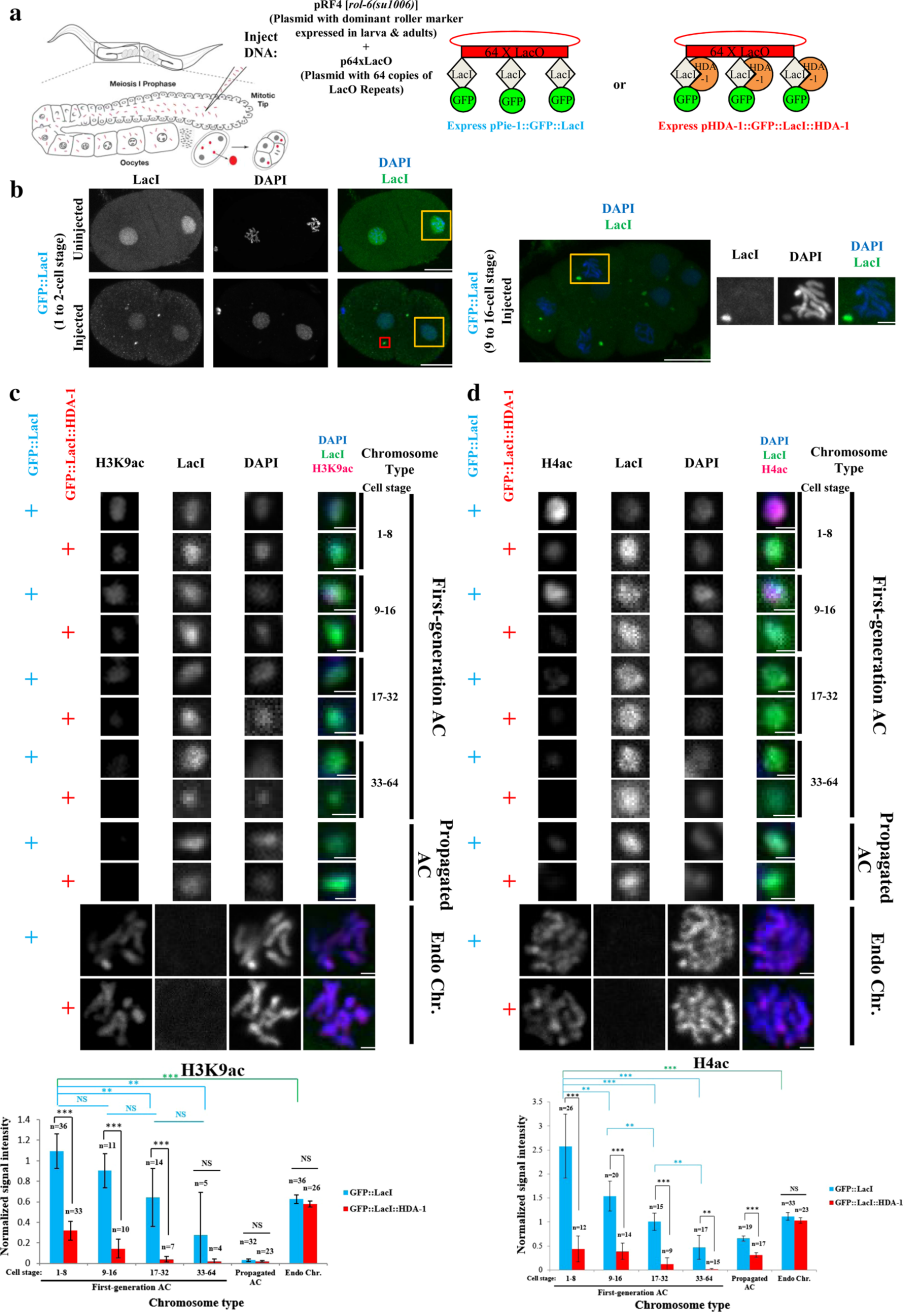
Newly formed ACs in *C. elegans* are enriched with histone H3K9 and H4 acetylations, which decline gradually in several embryonic cell divisions. **a** A schematic diagram of the experimental setup. Plasmid DNA with 64 copies of LacO repeats (p64xLacO) and a dominant roller mutant allele which express in larva and adults {pRF4[*rol*-*6*(*su1006*)]} was mixed and co-injected into strains expressing either GFP::LacI (under *pie*-*1* promoter) or GFP::LacI::HDA-1 (under *hda*-*1* promoter). Injected DNA concatemerized to form artificial chromosomes (ACs) in 1-cell embryos produced by injected worms. **b** On the left, a representative image of immunofluorescence of LacI (green) and DAPI (blue) is shown separately and merged in 1 or 2-cell stage embryos in GFP::LacI-tethering strain without or with DNA injection. Scale bar represents 10 μm. Examples of endogenous chromosomes in the nucleus are highlighted in yellow rectangles. LacI staining is nuclear in the uninjected case and forms foci and with weaker nuclear staining in the injected case. A representative AC is highlighted in a red rectangle in the injected case. On the right, a representative merged image of immunofluorescence of LacI (green) and DAPI (blue) in a 9 to 16-cell stage embryo in GFP::LacI-tethering strain after DNA injection is shown. Scale bar represents 10 μm. A cell with an AC is highlighted in a yellow rectangle, and magnified on the right with channels shown separately and merged, in which scale bar represents 2 μm. **c** Immunofluorescence of H3K9ac on first-generation ACs at different cell stages, and ACs that have been propagated for generations and endogenous chromosomes in GFP::LacI- and GFP::LacI::HDA-1-tethering strains. Cropped images containing ACs and endogenous chromosomes (Endo Chr.) were shown. Embryos were stained with antibody against H3K9ac (red), antibody against LacI (green) and DAPI (blue), shown separately and merged. Scale bar represents 1 μm for both ACs and endogenous chromosomes. Quantification of IF signals. Mean-corrected histone H3K9ac signal in each ROI was normalized with mean-corrected DAPI signal in each ROI, and the average normalized histone H3K9ac signal intensity was calculated. The number of cells (*n*) analyzed was indicated. Error bars indicate 95% confidence interval (CI) for the mean. ***p < 0.001 and **p < 0.01 by Student’s *t* test. NS means not significant. Black arcs show comparisons between GFP::LacI- and GFP::LacI::HDA-1-tethering strains at the same cell stage. Blue arcs show comparisons between ACs at different stages in GFP::LacI-tethering strain. Green arc shows a significant difference between 1 and 8-cell stage first-generation ACs and endogenous chromosomes in GFP::LacI-tethering strain. **d** Immunofluorescence of H4ac on first-generation ACs at different cell stages, and ACs that have been propagated for generations and endogenous chromosomes in GFP::LacI- and GFP::LacI::HDA-1-tethering strains. Cropped images containing ACs and endogenous chromosomes (Endo Chr.) were shown. Embryos were stained with antibody against H4ac (red), antibody against LacI (green) and DAPI (blue), shown separately and merged. Scale bar represents 1 μm for both ACs and endogenous chromosomes. Quantification of IF signals. Mean corrected histone H4ac signal in each ROI was normalized with DAPI signal in each ROI, and the mean normalized histone modification signal intensity was calculated. The number of cells (*n*) analyzed was indicated. Error bars indicate 95% confidence interval (CI) for the mean. ***p < 0.001 and **p < 0.01 by Student’s *t* test. NS means not significant. Black arcs show comparisons between GFP::LacI- and GFP::LacI::HDA-1-tethering strains at the same cell stage. Blue arcs show comparisons between ACs at different stages in GFP::LacI-tethering strain. Green arc shows a significant difference between 1 and 8-cell stage first-generation ACs and endogenous chromosomes in GFP::LacI-tethering strain

In order to decipher whether the role of histone modifications on de novo centromere formation in HACs is conserved in *C. elegans* ACs, we first studied the abundance of euchromatin-related histone modifications, H3K9 and H4 acetylations on newly formed, first-generation LacO-containing ACs in embryos. To generate visually trackable ACs, purified plasmid DNA containing 64 copies of LacO (p64xLacO) and a dominant roller marker expressed in larva and adults {pRF4 [*rol*-*6*(*su1006*)]} [38] was injected into the gonad of a worm strain expressing nuclear GFP::LacI and mCherry::H2B (Fig. 1a and Additional file 1: Fig. S1) [34]. The injected DNA concatemerizes and forms ACs in embryos produced by the injected worm. ACs containing LacO repeats are bound by GFP::LacI and can be observed as foci by live imaging of GFP or immunofluorescence using antibody against LacI (Fig. 1b). mCherry::H2B or DAPI signal was used to follow all chromosomes and determine the cell cycle stage of embryonic cells by live imaging and immunofluorescence, respectively. Consistent with our previous study [34], GFP::LacI foci representing ACs start to appear in 1-cell embryonic stage. These newly formed ACs are cytoplasmic in 1-cell stage and become nuclear in later cell stage (Fig. 1b) [34]. These newly formed ACs appear to be chromatinized at 1-cell stage already based on the co-localization of GFP::LacI and mCherry::H2B [34]. The level of histone modifications on ACs and endogenous chromosomes were compared in early embryos (from 1 to 64-cell stage) (Fig. 1c, d), during which most ACs gain their segregation ability [34]. By immunofluorescence, we found that H3K9ac and H4K5/8/12/16ac, both marks of relaxed chromatin, are significantly enriched on first-generation LacO-containing ACs at 1 to 8-cell stage, when compared to those on endogenous chromosomes (which do not change at different cell stages) (Fig. 1c, d and Additional file 2: Fig. S2). Interestingly, both marks gradually decline from 1 to 64-cell stage. The drop in H3K9 and H4 acetylation levels on new ACs over time is not due to a reduction in total H3 or H4 levels (Additional file 3: Fig. S3).

In heritable ACs that have been propagated for multiple generations, the acetylation levels are very low (and they do not vary at different cell stages) (Fig. 1c, d and Additional file 2: Fig. S2). This result is consistent with the findings that repetitive propagated ACs are enriched with H3K9me3 (Additional file 4: Fig. S4) [34]. Interestingly, the reduction in acetylation levels on propagated ACs in GFP::LacI::HDA-1-tethering strain is more severe than that in GFP::LacI-tethering strain (Fig. 1c, d), possibly due to the deacetylase activity of the HDA-1.

### Reducing H3K9ac/H4ac on newly formed ACs in *C. elegans* delays their ability to achieve accurate segregation

To investigate whether the histone acetylation on ACs plays a role in de novo centromere formation, we tethered HDA-1, a histone deacetylase expressed in *C. elegans* embryos [39–41], specifically to LacO-containing ACs by constructing a strain expressing GFP::LacI::HDA-1 under *hda*-*1* endogenous promoter (Additional file 8: Table 1). To verify the function of GFP::LacI::HDA-1 on ACs and confirm that it does not affect histone acetylation on endogenous chromosomes, immunofluorescence was performed to detect H3K9ac and H4ac levels in the GFP::LacI::HDA-1-tethering strain, and compared to those in the GFP::LacI-tethering strain and the enzymatically dead GFP::LacI::HDA-1(H145A)-tethering strain, in which HDA-1 has a point mutation at the deacetylase domain [42]. Both H3K9ac and H4ac levels on newly formed LacO ACs in 1 to 32-cell stage on GFP::LacI::HDA-1-tethering strain are significantly lower than those on GFP::LacI-tethering strain (Fig. 1c and d). On the other hand, H3K9ac and H4ac levels on new ACs tethered with GFP::LacI::HDA-1(H145A) are comparable to those tethered with GFP::LacI alone (Additional file 5: Fig. S5). This shows that the effect on ACs is specific to the deacetylase enzymatic activity of HDA-1 and not just due to the bulky tethering. Moreover, both H3K9ac and H4ac levels on endogenous chromosomes of GFP::LacI::HDA-1-tethering embryos have not decreased significantly, when compared to endogenous chromosomes of GFP::LacI-tethering embryos (Fig. 1c, d), indicating that GFP::LacI::HDA-1 are able to target LacO ACs specifically to decrease proximal histone acetylations without significantly affecting histone acetylation level on endogenous chromosomes.

**Fig. 2:**
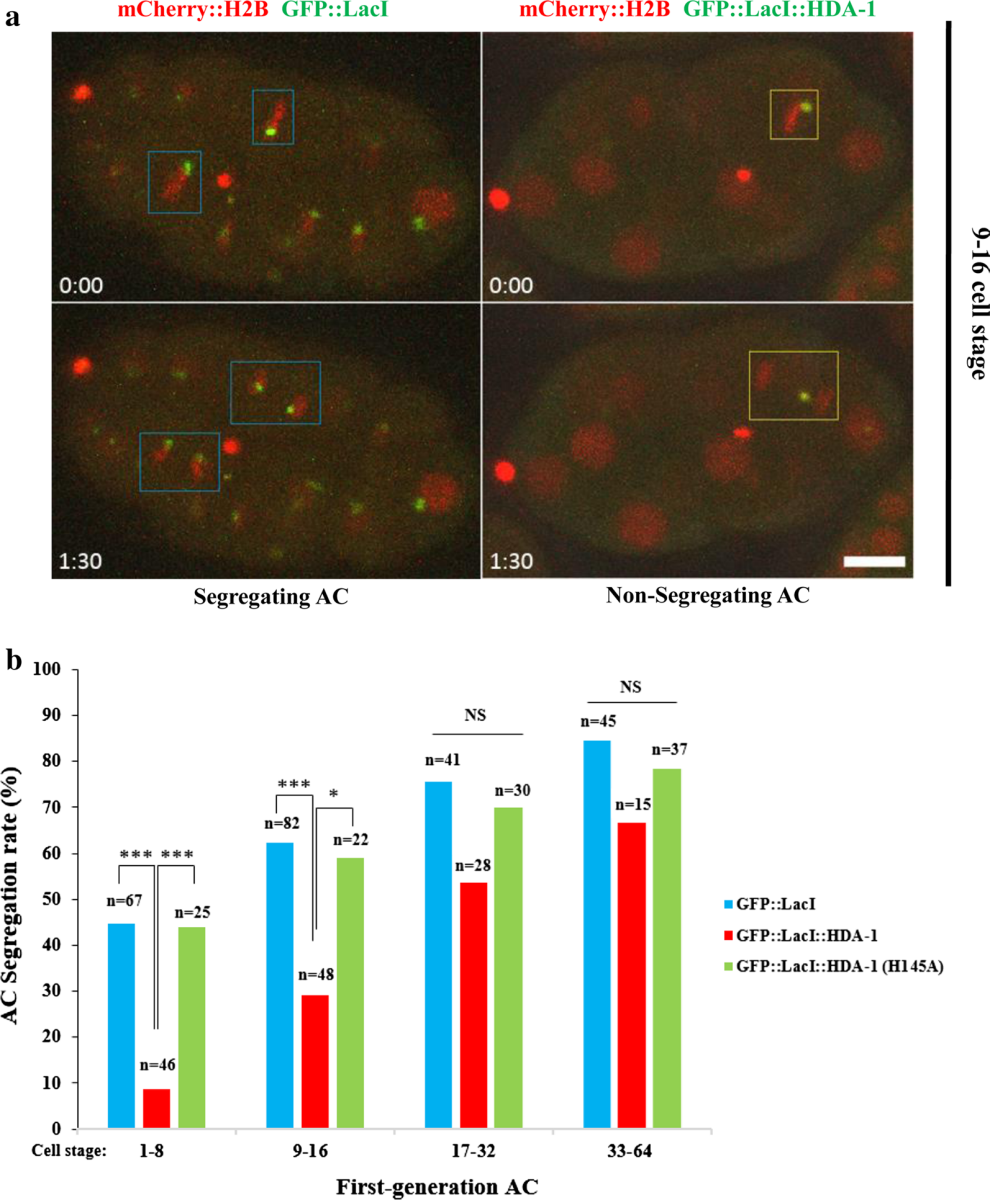
Histone H3K9 and H4 acetylations are important for rapid acquisition of segregation ability on newly formed ACs. **a** Representative live-cell images of GFP::LacI (green) and mCherry::H2B (red) in equally segregating ACs in GFP::LacI-tethering strain (left) and non-segregating ACs in GFP::LacI::HDA-1-tethering strain (right). In the left, LacO ACs aligned with metaphase plates (blue rectangles, top left) in a 9 to 16-cell stage GFP::LacI-tethering embryo. The ACs then segregated with endogenous chromosomes in anaphase (blue rectangles, bottom left). In the right, the LacO AC was adjacent to the metaphase plate but did not align with the metaphase plate (yellow rectangle, top right) in a 9 to 16-cell stage GFP::LacI::HDA-1-tethering embryo. The AC did not segregate with endogenous chromosomes in anaphase and was passively passed on to one of the daughter cells in anaphase (yellow rectangle, bottom right). The time lapse between the two images was shown (m:ss). Scale bar represents 10 μm. **b** Quantification of AC segregation rates in GFP::LacI-, GFP::LacI::HDA-1- and GFP::LacI::HDA-1(H145A)-tethering embryos. AC segregation rates are scored as the % of cells with segregating ACs among all dividing cells containing ACs. AC segregation rates in 1 to 8-, and 9 to 16-cell stages are significantly decreased in GFP::LacI::HDA-1-tethering strain when compared to GFP::LacI- and GFP::LacI::HDA-1(H145A)-tethering strains. The number of cells (*n*) analyzed was indicated. ***p < 0.001 and *p < 0.05 by Chi-squared test. Black arcs show comparisons among GFP::LacI-, GFP::LacI::HDA-1- and GFP::LacI::HDA-1(H145A)-tethering strains at the same cell stage

To track the segregation behavior of these newly formed ACs, we followed individual ACs by live-cell imaging, and quantified equal AC segregation events in GFP::LacI-, GFP::LacI::HDA-1-, and GFP::LacI::HDA-1(H145A)-tethering strains (Fig. 2). In each cell cycle, an AC without a functional centromere stays in the nucleus or cytoplasm and is passively transmitted to one of the daughter cells and may be lost [34]. On the contrary, an AC may align at the metaphase plate, independent of endogenous chromosomes, and segregate equally along with the sister chromatids during anaphase. Thus, equal AC segregation functionally implies that these ACs undergo de novo centromere formation and possesses a functional centromere. Consistent with our previous study [34], LacO ACs in the GFP::LacI-tethering strain rapidly gain segregation ability over a few cell cycles after their formation in 1-cell stage. Specifically, when the embryo reached 64-cell stage, 84% of mitotic cells containing ACs in the GFP::LacI-tethering strain contain at least one equally segregating AC (Fig. 2b). Remarkably, AC segregation capability is perturbed in early embryos in the GFP::LacI::HDA-1-tethering strain, specifically from 1 to 16-cell stage. AC segregation rates decrease by 5.3 and twofold in 1 to 8-cell and 9 to 16-cell stages, respectively. Consistent with the H3K9ac and H4ac level, GFP::LacI::HDA-1(H145A)-tethering does not affect the AC segregation frequency (Fig. 2b). This result suggests that the high levels of H3K9ac and H4ac on new ACs enable efficient acquisition of accurate AC segregation, in which the effects are more prominent in early cell divisions.

### Histone H3K9 and H4 acetylations facilitate the initial CENP-A^HCP−3^ and NDC-80 deposition on the de novo centromere on newly formed ACs

**Fig. 3:**
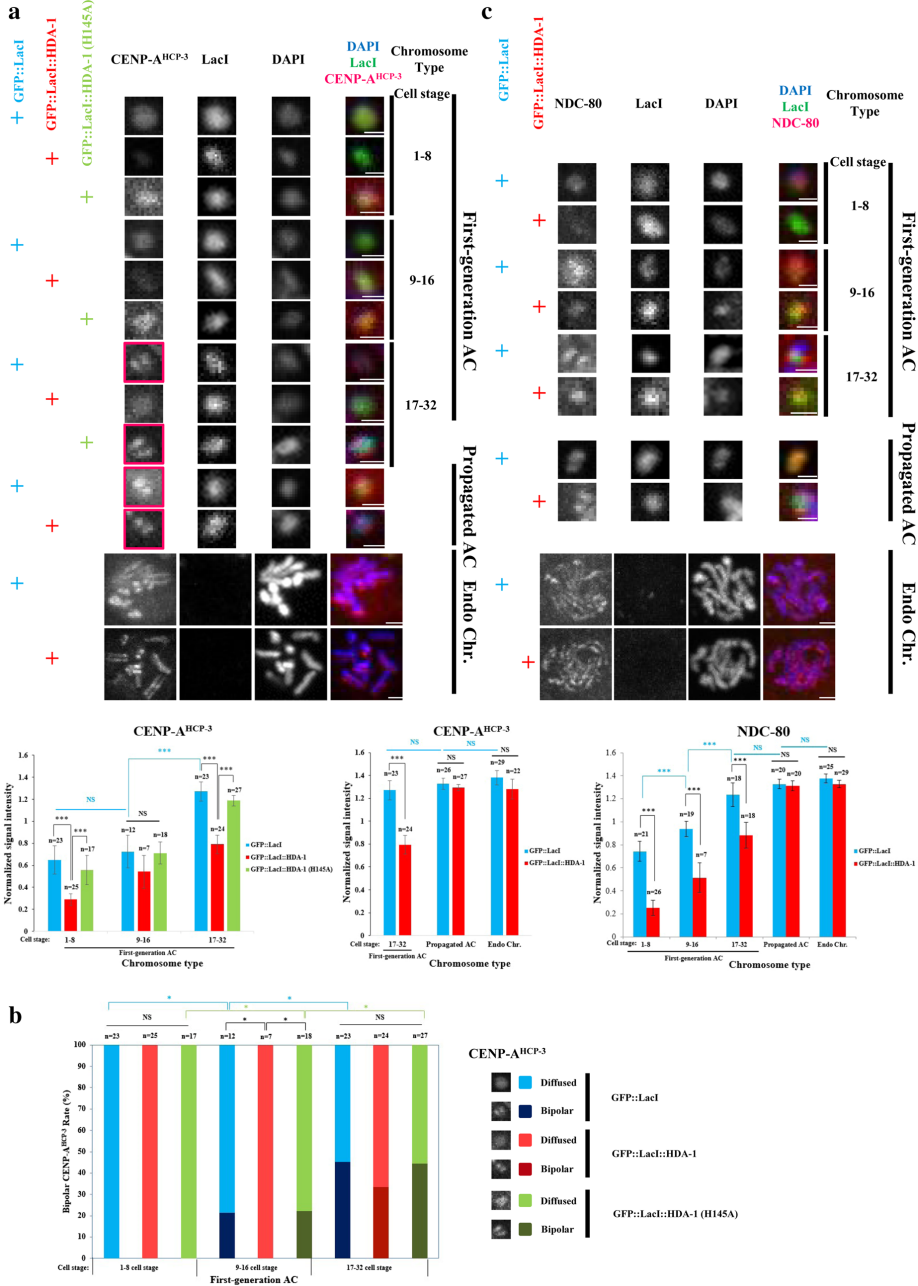
Histone H3K9 and H4 acetylations facilitate initial CENP-A^HCP−3^ and NDC-80 deposition on newly formed ACs. **a** Immunofluorescence of CENP-A^HCP−3^ on first-generation ACs at different cell stages, and ACs that have been propagated for generations and endogenous chromosomes in GFP::LacI-, GFP::LacI::HDA-1- and GFP::LacI::HDA-1(H145A)-tethering strains. Cropped images containing ACs and endogenous chromosomes (Endo Chr.) were shown. Embryos were stained with antibody against CENP-A^HCP−3^ (red), antibody against LacI (green) and DAPI (blue), shown separately and merged. Scale bar represents 1 μm for both ACs and endogenous chromosomes. Examples of bipolar-oriented CENP-A^HCP−3^ are highlighted in red boxes. Quantification of IF signals. CENP-A^HCP−3^ signals were normalized with DAPI signals, and the average normalized CENP-A^HCP−3^ signal intensity was calculated. The number of cells (*n*) analyzed was indicated. Error bars indicate 95% confidence interval (CI) for the mean. ***p < 0.001 by Student’s *t* test. NS means not significant. Black arcs show comparisons between GFP::LacI-, GFP::LacI::HDA-1- and GFP::LacI::HDA-1(H145A)-tethering strain at the same cell stage. Blue arcs show comparisons between ACs at different stages in GFP::LacI-tethering strain. **b** Quantification of the % of cells with bipolar or diffused CENP-A^HCP−3^ on the newly formed ACs in 1 to 8-, 9 to 16- and 17 to 32-cell stage in GFP::LacI-, GFP::LacI::HDA-1- and GFP::LacI::HDA-1(H145A)-tethering strains. The number of cells (*n*) analyzed was indicated. *p < 0.05 by Chi-squared test. Black arcs show comparisons among GFP::LacI-, GFP::LacI::HDA-1- and GFP::LacI::HDA-1(H145A)-tethering strains at the same cell stage. Blue arcs show comparisons between ACs at different stages in GFP::LacI-tethering strain. Green arcs show comparisons between ACs at different stages in GFP::LacI::HDA-1(H145A)-tethering strain. **c** Immunofluorescence of NDC-80 on first-generation ACs at different cell stages, and ACs that have been propagated for generations and endogenous chromosomes in GFP::LacI- and GFP::LacI::HDA-1-tethering strains. Cropped images containing ACs and endogenous chromosomes (Endo Chr.) were shown. Embryos were stained with antibody against NDC-80 (red), antibody against LacI (green) and DAPI (blue), shown separately and merged. Scale bar represents 1 μm for both ACs and endogenous chromosomes. Quantification of IF signals. NDC-80 signals were normalized with DAPI signals, and the average normalized NDC-80 signal intensity was calculated. The number of cells (*n*) analyzed was indicated. Error bars indicate 95% confidence interval (CI) for the mean. ***p < 0.001 by Student’s *t* test. NS means not significant. Black arcs show comparisons between GFP::LacI- and GFP::LacI::HDA-1-tethering strains at the same cell stage. Blue arcs show comparisons between ACs at different stages in GFP::LacI-tethering strain

To determine whether histone acetylations affect the assembly of new centromeres, we analyzed the level of CENP-A^HCP−3^ and outer kinetochore protein NDC-80 on GFP::LacI- and GFP::LacI::HDA-1-tethered newly formed ACs by immunofluorescence (Fig. 3a, c). At comparable embryo stages, ACs with GFP::LacI-tethering contain more CENP-A^HCP−3^ and NDC-80 than those with GFP::LacI::HDA-1-tethering. This result suggests that histone acetylations facilitate the initial deposition of CENP-A^HCP−3^ and other kinetochore proteins on newly formed ACs.

Interestingly, the amount of CENP-A^HCP−3^ and NDC-80 signals on newly formed ACs increased with embryonic divisions (Fig. 3a, c), consistent with the rise in segregation frequency over time (Fig. 2), suggesting that the de novo kinetochore becomes more mature and functional over time. Indeed, the average signal intensity of CENP-A^HCP−3^ and NDC-80 on 17 to 32-cell stage first-generation ACs in GFP::LacI-tethering strains becomes comparable to those on propagated ACs and endogenous chromosomes, which segregate equally (Fig. 3a). The difference in segregation frequency and CENP-A^HCP−3^ between these two strains can only be observed in first-generation ACs at early embryo stage. In consistence with the signal increase of CENP-A^HCP−3^ on the newly formed ACs over time, CENP-A^HCP−3^ orientation changes from diffuse (100% in 1 to 8-cell stage) to bi-oriented (45% in 17 to 32-cell stage) in GFP::LacI-tethering strain (Fig. 3b). The mechanism behind this natural maturation of centromere in GFP::LacI-tethering strain is not completely clear.

### Histone acetylations are correlated with active transcription on newly formed ACs during centromere formation, but transcription is not required for maintenance of centromere

**Fig. 4:**
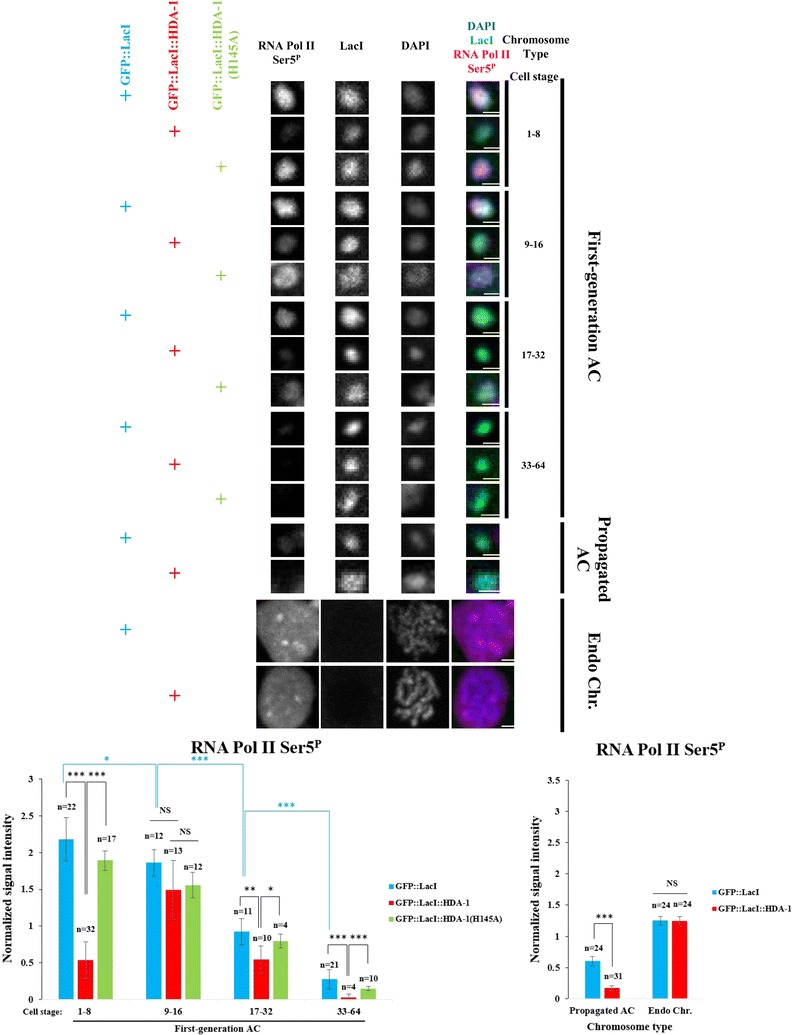
Histone deacetylase HDA-1 tethering reduces RNA polymerase II-mediated transcription initiation signal on newly formed ACs. Immunofluorescence of RNA polymerase II Serine 5 phosphorylation (RNA Pol II Ser5^P^) on first-generation ACs at different cell stages, and ACs that have been propagated for generations and endogenous chromosomes in GFP::LacI-, GFP::LacI::HDA-1- and GFP::LacI::HDA-1(H145A)-tethering strain. Cropped images containing ACs and endogenous chromosomes (Endo Chr.) were shown. Embryos were stained with antibody against RNA Pol II Ser5^P^ (red), antibody against LacI (green) and DAPI (blue), shown separately and merged. Scale bar represents 1 μm for both ACs and endogenous chromosomes. Quantification of IF signals. RNA Pol II Ser5^P^ signals were normalized with DAPI signals, and the mean normalized RNA Pol II Ser5^P^ signal intensity was calculated. The number of cells (*n*) analyzed was indicated. Error bars indicate 95% confidence interval (CI) for the mean. ***p < 0.001, **p < 0.01 and *p < 0.05 by Student’s *t* test. NS means not significant. Black arcs show comparisons between GFP::LacI- and GFP::LacI::HDA-1-tethering strains at the same cell stage. Blue arcs show comparisons between ACs at different stages in GFP::LacI-tethering strain

To investigate whether H3K9ac and H4ac facilitate initial CENP-A^HCP−3^ loading through transcription, we examined RNA polymerase II largest subunit (RPB1) C-terminal domain repeat (YSPTSPS) Ser5 phosphorylation (Ser5^P^), which promotes transcription initiation [43–45], on newly formed ACs at different embryo stages, and on the endogenous chromosomes in GFP::LacI-, GFP::LacI::HDA-1-, and GFP::LacI::HDA-1(H145A)-tethering strains (Fig. 4). In GFP::LacI-tethering strain, while most RNA Pol II Ser5^P^ signal surrounds endogenous chromosomes in prometaphase at different embryo stages, the Ser5^P^ signal is very strong on the newly formed ACs at 1 to 8-cell stages, but then declines gradually. Tethering GFP::LacI::HDA-1 to ACs reduces their RNA Pol II Ser5^P^ signal strongly at 1 to 8-cell stage, and to a lesser extent at 17 to 64-cell stage, but does not affect that on endogenous chromosomes (Fig. 4). As expected, tethering GFP::LacI::HDA-1(H145A) has no significant effect on new ACs’ RNA Pol II Ser5^P^ signal. These results suggest that high histone acetylation levels at H3K9 and H4K5/8/12/16ac can induce transcription initiation. Interestingly, H3K4me2, a mark that implicates active transcription, is not detected on 1 to 8-cell stage GFP::LacI-tethered ACs, but it increases in 9 to 16-cell stage, and then gradually declines as for the acetylated marks and Ser5^P^ (Additional file 6: Fig. S6). On propagated ACs, the RNA Pol II Ser5^P^ signal is relatively low, consistent with H3K9me3 accumulation (Additional file 4: Fig. S4) [34] and gene silencing observed on propagated, repetitive ACs [46].

**Fig. 5:**
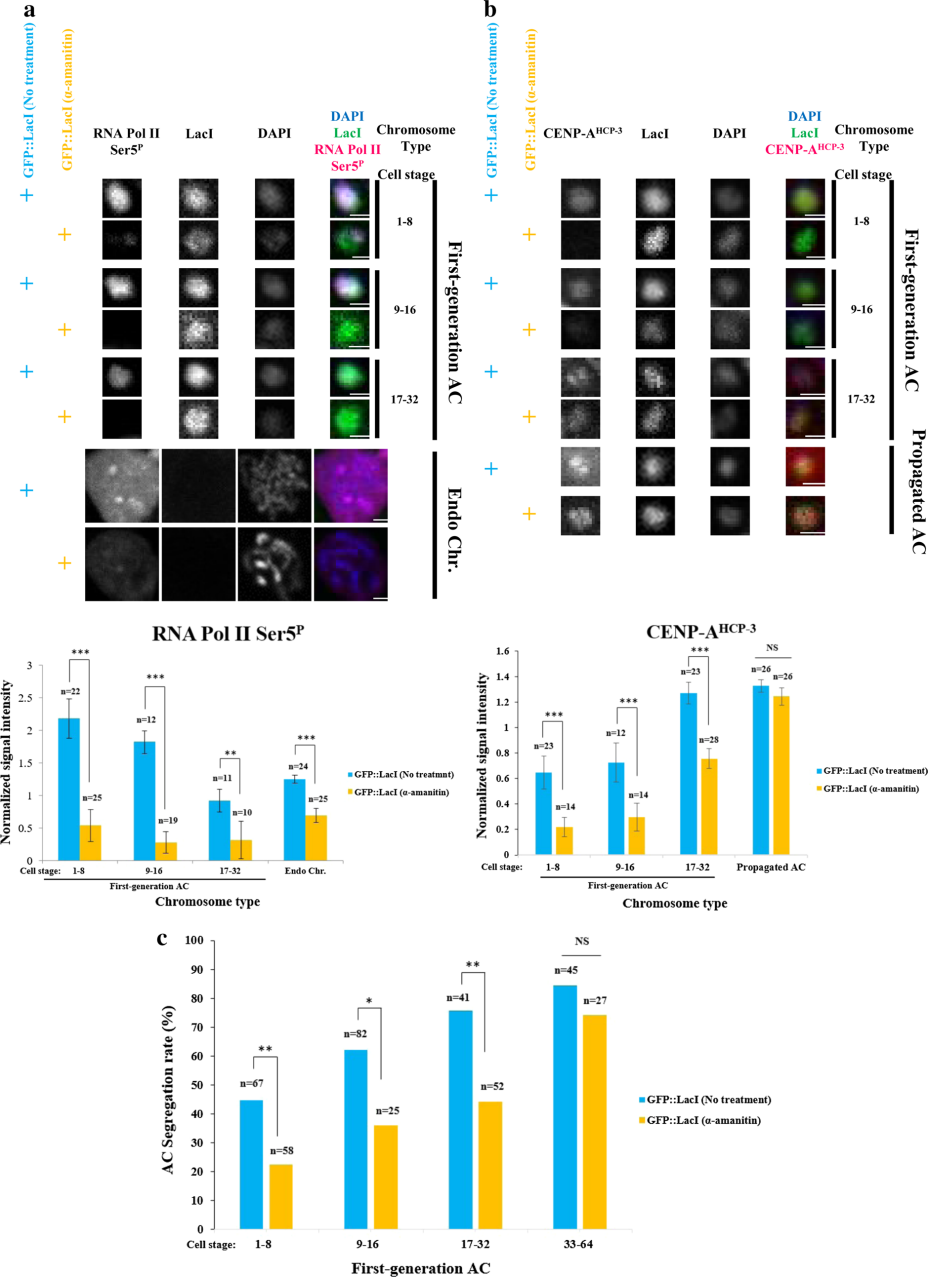
RNA polymerase II-mediated transcription initiation is important for initial CENP-A^HCP−3^ deposition on newly formed ACs. **a** Immunofluorescence of RNA polymerase II Serine 5 phosphorylation (RNA Pol II Ser5^P^) on first-generation ACs at different cell stages, and endogenous chromosomes in GFP::LacI-tethering strain without and with alpha-amanitin treatment. Cropped images containing ACs and endogenous chromosomes (Endo Chr.) were shown. Embryos were stained with antibody against RNA Pol II Ser5^P^ (red), antibody against LacI (green) and DAPI (blue), shown separately and merged. Scale bar represents 1 μm for both ACs and endogenous chromosomes. Quantification of IF signals. RNA Pol II Ser5^P^ signals were normalized with DAPI signals, and the average normalized RNA Pol II Ser5^P^ signal intensity was calculated. The number of cells (*n*) analyzed was indicated. Error bars indicate 95% confidence interval (CI) for the mean. ***p < 0.001 and **p < 0.01 by Student’s *t* test. NS means not significant. Black arcs show comparisons between no treatment and alpha-amanitin treatment at the same cell stage. The data for GFP::LacI-tethering strain without alpha-amanitin treatment are the same as in Fig. 4. **b** Immunofluorescence of CENP-A^HCP−3^ on first-generation ACs at different cell stages, and ACs that have been propagated for generations in GFP::LacI-tethering strain without and with alpha-amanitin treatment. Embryos were stained with antibody against CENP-A^HCP−3^ (red), antibody against LacI (green) and DAPI (blue), shown separately and merged. Scale bar represents 1 μm. Quantification of IF signals. CENP-A^HCP−3^ signals were normalized with DAPI signals, and the average normalized CENP-A^HCP−3^ signal intensity was calculated. The number of cells (*n*) analyzed was indicated. Error bars indicate 95% confidence interval (CI) for the mean. ***p < 0.001 by Student’s *t* test. NS means not significant. Black arcs show comparisons between no treatment and alpha-amanitin treatment at the same cell stage. The data for GFP::LacI-tethering strain without alpha-amanitin treatment are the same as in Fig. 3a. **c** Quantification of AC segregation rates in GFP::LacI-tethering strain without and with alpha-amanitin treatment embryos. AC segregation rates are scored as the % of cells with segregating ACs among all dividing cells containing ACs. The number of cells (*n*) analyzed was indicated. *p < 0.05 and **p < 0.01 by Chi-squared test. Black arcs show comparison between without and with alpha-amanitin treatment at the same cell stage

To test if transcription is required for initial CENP-A^HCP−3^ deposition, we applied RNA polymerase II and III inhibitor alpha-amanitin to *perm*-*1* RNAi-treated, permeabilized GFP::LacI-tethering embryos to inhibit transcription, and monitor the level of RNA Pol II Ser5^P^, histone acetylations, CENP-A^HCP−3^ and AC segregation frequency (Fig. 5 and Additional file 7: Fig. S7). We found that alpha-amanitin treatment globally reduces RNA Pol II Ser5^P^ on all stages of new ACs and endogenous chromosomes (Fig. 5a) and slows down the accumulation of CENP-A^HCP−3^ on new ACs (Fig. 5b), inhibiting equal AC segregation in 1 to 32-cell stage (Fig. 5c). These results show that inhibiting transcription, similar to histone deacetylation, has a delaying effect on de novo centromere establishment. Reciprocally, alpha-amanitin reduces H3K9ac and H4ac levels on new ACs specifically in early embryo cell stages, and on endogenous chromosomes  (Additional file 7: Fig. S7).

## Discussion

In this study, we investigated the relationship between histone modifications, transcription and de novo centromere establishment. We suggest that in order for a de novo centromere to efficiently form and function, two categories of epigenetic mark or histone modifications may be required. The first ones are seeding marks that favor incorporation of CENP-A nucleosomes or other centromeric proteins into ectopic, originally non-centromeric regions. Such marks may help to establish a de novo centromere identity. Once established, the second group of epigenetic marks may reinforce the centromere identity and propagates the de novo centromere through mitosis and meiosis, such as histone modifications that help with centromere licensing and new CENP-A deposition during every cell cycle. The histone modifications that propagate endogenous existing centromeres and stably maintained de novo centromeres appear to be the same [29, 30]. However, histone modifications affecting de novo centromere establishment are less clear. A previous study in HACs has found that euchromatin mark H3K9ac promotes de novo centromere formation, whereas the heterochromatin mark H3K9me3 antagonizes de novo centromere formation [32]. Interestingly, once the de novo centromere is established, H3K9ac is no longer needed [32].

To seed a new centromere, CENP-A or other epigenetic marks of the centromere must be ectopically incorporated into non-existing centromeric chromatin. No matter whether these epigenetic marks are deposited by a chaperone, histone modifier or incorporated autonomously, an open chromatin will be more accessible to these proteins. We hypothesized that an open chromatin may be advantageous for new CENP-A incorporation in a replication-independent manner. Although both H3/4 acetylations and H3K4me2 are open chromatin marks associated with active transcription [47, 48], they may influence chromatin structure through different mechanisms. H3K4me2 allows binding of specific transcription factors or chromatin remodelers to influence transcription [49–51]. H3/4 acetylations open the chromatin structure by adding an acetyl group to lysine to remove its positive charge, thereby loosening the interaction between histones and the wrapping DNA, and resulting in increased histone turnover [52–55]. Moreover, H3/4 acetylations have the ability to recruit specific proteins, such as bromodomain-containing proteins, as histone readers to the modified chromatin [56–58], and allow transcription factors and RNA polymerase to bind to mediate transcription. H3/4 acetylations also have broader chromatin functions, such as in DNA replication and DNA damage repair [59, 60]. Thus, open chromatin marks such as H3/4 acetylations may contribute to efficient de novo centromere formation by facilitating nucleosome disassembly and assembly, and initial CENP-A incorporation (Fig. 3). Alternatively, acetylation on newly synthesized histones, such as H4K5/12ac, may affect the nuclear transport and deposition of soluble CENP-A-H4 tetramers [61]. Based on this hypothesis, a previous HAC study has shown the role of H3K9 acetylation using a HAC formation assay by a 6-week selection process followed by fluorescence in situ hybridization (FISH)-immunofluorescence analysis [32]. In consistence, we detected hyperacetylations on H3K9 and H4 in early (1 to 8-cell stage), first-generation LacO-containing ACs formed in *C. elegans* embryos (Fig. 1c, d). We further showed that H3/4 hyperacetylations on ACs contribute to its rapid segregation capability (Fig. 2) and de novo centromere formation (Fig. 3) in a whole organism model.

**Fig. 6:**
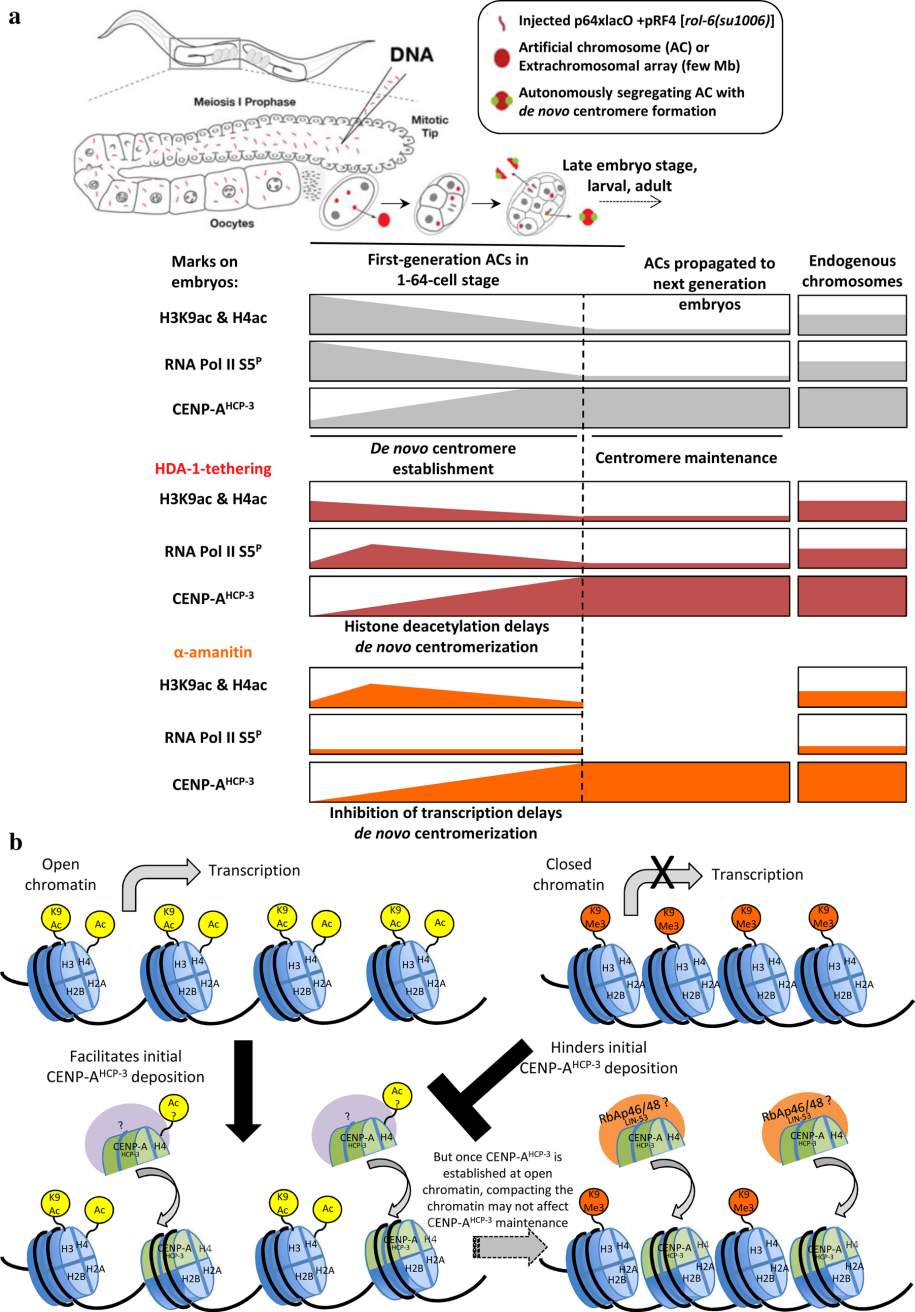
Summary and model of the effects of HDA-1-tethering and transcription on de novo centromere establishment. **a** Summary of the levels of H3K9 and H4 acetylations, RNA polymerase II serine 5 phosphorylation, and CENP-A^HCP−3^ in first-generation ACs at progressing embryo stages, propagated ACs and endogenous chromosomes in GFP::LacI- or GFP::LacI::HDA-1-tethering strains, and in GFP::LacI-tethering strain with alpha-amanitin treatment. **b** Schematic model of the proposed mechanism of histone acetylations and transcription in facilitating initial CENP-A^HCP−3^ incorporation into newly formed ACs, and supporting de novo centromere formation

The hyperacetylations on H3K9 and H4 in early, first-generation LacO-containing ACs formed in *C. elegans* embryos decline over several divisions (Fig. 1c, d). Based on the HAC results [32], H3K9ac may only be required for the establishment of de novo centromeres but not for their maintenance. We previously showed that HP1, which binds to the heterochromatin mark H3K9me3, hinders de novo centromere formation [34]. Indeed, H3K9me3 is not apparent in first-generation ACs, while stably transmitted, repetitive ACs that were propagated through multiple generations in *C. elegans* accumulate H3K9me3 (Additional file 4: Fig. S4) [34]. Together, our studies suggest that newly formed ACs in early embryo stage are hyperacetylated at H3K9, but are deacetylated and trimethylated after de novo centromeres are well established (Figs. 1c, 6a), though the endogenous enzyme for the initial acetylation and subsequent deacetylation is not clear.

Although H3/4 acetylations contribute to de novo centromere formation based on our study and the HAC study [32], whether they function in centromere maintenance is less clear. Previous studies have shown that H3/4 hypoacetylation occur in human and *Drosophila* endogenous centromeric region [9], as well as in *S. pombe* centromeric inner and outer repeats [62–64]. The heterochromatin and histone hypoacetylation flanking the regional centromere, such as those present in *S. pombe* outer repeats, are thought to recruit cohesin to keep sister chromatids together [65]. In *C. elegans*, the heterochromatin is at the arms of the chromosomes but not exactly flanking the holocentromeres, so the role of heterochromatin and hypoacetylation in holocentromere function or cohesion remains unclear. Recently, endogenous human centromeres are shown to be acetylated temporarily before new CENP-A is deposited [32, 64]. Tethering of a histone acetyltransferase even partially rescued the CENP-A assembly defect on established HACs in hMis18α depletion [32, 54]. In *C. elegans*, RbAp46/48^LIN−53^, known to associate with HAT-1^TAG−235^ [66], is required for CENP-A assembly on endogenous holocentric chromosomes, but HAT-1^TAG−235^ depletion does not affect endogenous chromosome segregation [25]. In contrast to de novo centromeres, existing, endogenous centromeres do not require ectopic CENP-A or other centromeric proteins to be incorporated into non-centromeric chromatin. Open chromatin marks that are advantageous for the initial deposition of centromeric proteins onto de novo centromeres on ACs may not be required for stably maintained centromeres (Fig. 6b). Other mechanisms, including the involvement of CENP-A chaperones, may be sufficient to propagate the existing centromeres.

Transcription is associated with nucleosome remodeling and chromatin modification processes. For instance, some transcriptional adaptor proteins containing the acetyltransferase subunit can acetylate histone H3 and H4 at specific lysines by to make the chromatin more accessible for gene activation [67, 68]. Alternatively, acetylation of soluble H4 may stimulate the initial deposition of CENP-A^HCP−3^-H4 nucleosomes [61]. In humans, centromeres are transcribed during G1 phase [69], coinciding with the time of CENP-A deposition. Centromeric transcription may couple with nucleosome exchange to incorporate CENP-A nucleosomes into centromeric, open chromatin [70–72]. In *C. elegans*, gene transcription has been shown to begin at 4-cell stage [73]. We detected transcription initiation on newly formed GFP::LacI-tethered ACs, especially at early embryo stages (Fig. 4). Inhibition of RNA Pol II reduces the level of CENP-A on newly formed ACs (Fig. 5b), and ACs equal segregation frequency (Fig. 5c). Histone deacetylation of newly formed ACs reduces transcription initiation on ACs, especially at 1 to 8-cell stage (Fig. 4), and reciprocally, inhibition of RNA Pol II reduces H3K9 and H4 acetylation on ACs (Additional file 7: Fig. S7), confirming the interdependent relationship between histone acetylation and transcription. These results suggest that transcription, together with histone acetylation, can epigenetically regulate de novo CENP-A^HCP−3^ loading and centromere formation. Surprisingly, CENP-A^HCP−3^ domains are indeed negatively correlated with RNA Pol II (*r* = − 0.66), H3K36me2/3 (*r* = − 0.6) and H3K4me2 (*r* = − 0.49) occupancy on endogenous *C. elegans* chromosomes [7, 23]. Moreover, transcription does not affect CENP-A^HCP−3^ maintenance on repetitive propagated ACs (Fig. 5b) and on endogenous chromosomes in *C. elegans* [7]. Such difference in the effect of transcription may be attributed to the mechanism of de novo centromere formation and centromere propagation of existing centromeres.

In human cells, HAC formation (up to 30% in HT1080 cell line) is identified after 6 weeks of selection, transformant isolation and confirmation of the presence of CENP-A on HACs without genomic integration [31, 32]. In *C. elegans*, an average of 3 ACs was observed in 1-cell stage embryos without further increase in later cell stage [34], suggesting that AC formation finishes by 1-cell stage, and enabling the tracing of a large population of individual ACs for analysis. By 64-cell stages (in 6 cell divisions, < 200 min since fertilization) [37], the majority of mitotic cells containing ACs (84%) contain ACs that can equally segregate. This provides an efficient model for de novo centromere formation. Moreover, any DNA sequence can be injected for AC formation in *C. elegans* [6, 7, 24, 34, 74], but alpha satellites must be transfected for HAC formation in human cells. The difference in de novo centromere formation rate in *C. elegans* and humans may be related to the plasticity nature of holocentromeres in *C. elegans*. The kinetochore protein complexes on holocentromeres are largely similar to those on monocentromeres [6, 34, 74]. No centromere-specific sequences have been identified in *C. elegans*, although GA-rich motifs, which coincide with transcription factor high occupancy target (HOT) sites, are common to the centromeric sites [24]. Besides, CENP-A^HCP−3^ domains are positively correlated with H3K27me3 (*r* = 0.64) and H3K9me3 (*r* = 0.44) [7, 23, 24]. These findings suggest that holocentromere regulation is largely epigenetic. Therefore, de novo centromere formation in holocentromeres may be more promiscuous than in monocentromeres.

Our study applied a straightforward functional assay to monitor de novo centromere formation. In addition to measuring the final stable AC formation rate as in HAC studies, we can also observe the change in AC segregation frequency in different embryo stages. By live-cell imaging of consecutive cell divisions in early embryos of *C. elegans*, we can directly observed a first-generation AC matures from undergoing passive inheritance in one division to equally segregating in the next mitosis, implicating a de novo centromere formation event [34]. Using this real-time method, we were able to pinpoint the formation time of each de novo centromere down to a particular cell cycle, and track the fate of the AC in subsequent divisions. Currently, how and why ACs matures over time naturally is still not fully understood. Albeit tethering of HDA-1 histone deacetylase or inhibiting transcription can delay the formation of de novo centromeres on ACs, they eventually can be matured and stably propagated (Fig. 6a). How CENP-A^HCP−3^ initially loads and form de novo centromere even in H3K9/H4 hypoacetylated environment remains an open question. The real-time observation approach without selection offers a valuable opportunity to better understand the mechanism underlying de novo centromere establishment in vivo. We can temporally monitor histone modifications, kinetochore protein levels and chromosome segregation at different embryo cell stages, from before the establishment of a functional centromere to after. In this study, we use a protein tethering approach based on LacO:LacI binding to specifically recruit a histone modifier to ACs, analogous to previous studies in HACs, where tetO sequences were inserted to centromeric alpha-satellite DNA [28–33]. In contrast to the application of a drug against a histone modifier, which affects the whole cell with pleiotropic effects, our genetic engineering method is intended not to affect the endogenous chromosome chromatin environment and thus is more specific.

Our study also demonstrates a direct way to manipulate the AC chromatin environment in order to study de novo centromere formation, which may help to design ACs that segregate more faithfully, a crucial factor for their use as cloning vectors. The size of the GFP foci binding to the ACs does not change through the embryo stages since 1-cell stage [34], suggesting the size of the ACs does not increase over time. However, one of the caveats in our cytological assay is that we were not able to isolate these newly formed, first-generation ACs for biochemical analyses, including chromatin immunoprecipitation (chIP) to investigate DNA–protein interactions. On the other hand, we could isolate stable, propagating ACs for chIP study. Live imaging and chIP analyses of ACs should complement each other to obtain high temporal resolution of centromere establishment dynamics, and high sequence resolution of de novo centromere architecture, respectively.

## Conclusions

Here, we showed that histone acetylations facilitate efficient de novo centromere formation. Moreover, our study implicated that the histone acetylation and transcription-driven de novo centromere establishment is not just an aberrant event that merely occurs in cancer cells or cell lines; rather, it can also occur in physiologically normal cells in whole organisms and thus is biologically relevant. Likely, a balance between de novo centromere formation and suppression is necessary for maintaining genome stability in development and evolution.

## Methods

### Worm strain construction and DNA injection

All strains were maintained at 20 °C. WYY7 (*unc*-*119(ed3)III; hkuSi2[hda*-*1p::GFP::LacI::hda*-*1; cb*-*unc*-*119(*+*)(WYYp33)]II; ltIs37 [pie*-*1p::mCherry::his*-*58 (pAA64) *+* unc*-*119(*+*)]*) was made by inserting a single copy of GFP::LacI::HDA-1 (genomic DNA with introns) into EG6699 using MosSCI [75, 76] and subsequently crossed with OD56 to obtain a double homozygous of GFP::LacI::HDA-1 and mCherry::H2B (Additional file 8: Table 1). The GFP::LacI::HDA-1 is regulated by 2.5 kb of endogenous *hda*-*1* promoter and a 0.6 kb of *hda*-*1* 3′ UTR sequence. WYY28 (*unc*-*119(ed3)III; hkuSi6[Phda*-*1:: GFP::LacI::hda*-*1(H145A)::hda*-*1 3′UTR; cb*-*unc*-*119(*+*)(WYYp112)]II; ltIs37 [pie*-*1p::mCherry::his*-*58 (pAA64) *+* unc*-*119(*+*)]*) was made by PCR site-directed mutagenesis of WYYp33 to mutate histidine at residue 145 to alanine (H145A), and constructed similarly to WYY7 (Additional file 8: Table 1). A mixture of purified p64xLacO plasmid (100 ng/μl) and pRF4 plasmid (50 ng/μl), carrying the *rol*-*6(su1006)* dominant marker, which gives a roller phenotype in larva and adults, was co-injected into gonad of young adult worms with standard procedure [34]. Worms were recovered at 20 °C for 4–8 h before subsequent live imaging or immunofluorescence of newly formed ACs. Propagated ACs were constructed by selecting F1 and next-generation worms that roll.

### Immunofluorescence

Worms were dissected on polylysine-coated glass slides in a droplet of M9 buffer. Samples were then flattened with a glass cover slip and freeze-cracked in liquid nitrogen. Samples were fixed in cold methanol for 30 min. They were stained with primary antibodies (0.93 mg/ml mouse monoclonal anti-LacI (Millipore 05-503), 1.16 mg/ml rabbit polyclonal anti-H3K4me2 (Novus Biologicals NB21-1022), 1 mg/ml rabbit polyclonal anti-H3K9ac (Millipore ABE18), 1 mg/ml rabbit polyclonal anti-H4ac, which recognizes H4K5ac, H4K8ac, H4K12ac and H4K16ac (Millipore 06-598), 1 mg/ml rabbit polyclonal anti-Histone H3 (Abcam ab1791), 1 mg/ml rabbit polyclonal anti-Histone H4 (Abcam ab10158), 1 mg/ml rabbit polyclonal anti-CENP-A^HCP−3^ (Novus Biologicals 29540002), 1 mg/ml rabbit polyclonal anti-NDC-80 (OD32, a gift from Arshad Desai) and 0.8 mg/ml rabbit polyclonal anti-RNA Polymerase II phospho Ser5 (Ser5^P^) (Abcam ab5131)) diluted 1:500 (or 1:1000 for CENP-A^HCP−3^ and NDC-80 antibodies) in Abdil buffer and then stained with 0.75 mg/ml fluorescence-conjugated secondary antibodies [anti-Rabbit IgG-Alexa 647 (Jackson Immuno Research 111-606-045) and anti-Mouse IgG-FITC (Jackson Immuno Research 115-096-062)] diluted 1:500 in PBST as previously described [34]. IF slides were imaged with a Carl Zeiss LSM780 laser scanning confocal microscope with a Plan-Apochromat 40× Oil objective and PMT detectors. Stacks with 25 × 0.5 μm planes were scanned for each embryo in a 4× zoom. DAPI, FITC and Alex647 channels were scanned with a 2.55 μs pixel dwell time and 36 μm pinhole for all 3 channels. The images were saved in 16 bits format. At least 2 independent experiments have been performed, using the same batch of antibody solution.

### Immunofluorescence signal quantification

To define the areas of measurement for each individual AC or the bulk endogenous chromosomes, a region of interest (IROI) enclosing the AC (based on LacI) or endogenous chromosomes was selected and a slightly larger region enclosing the ROI (LROI) was drawn. The total LacI, histone modification, CENP-A^HCP−3^, NDC-80, RNA Pol II Ser5^P^ or DAPI signal intensity was measured in the ROI (*I*_ROI_) and LROI (*I*_LROI_) for each AC and endogenous chromosomes. The mean background signal intensity (*i*_b_) was calculated by subtracting the total intensity in ROI from the total intensity in LROI and then divided by the area in between the two ROIs (*A*_LROI_ − *A*_ROI_) (i.e. *i*_b_ = (*I*_LROI_ − *I*_ROI_)/(*A*_LROI_ − *A*_ROI_)). The background signal intensity in ROI (*B*_ROI_) was calculated by multiplying the mean background signal intensity by the area in ROI (i.e. *B*_ROI_ = *i*_b_ × *A*_ROI_). The total corrected signal in ROI (*C*_ROI_) was calculated by subtracting the background signal in ROI from the signal in ROI (*C*_ROI_ = *I*_ROI_ − *B*_ROI_). The mean corrected signal (*c*_ROI_) was calculated by the total corrected signal in ROI divided by the area in ROI (i.e. *c*_ROI_ = *C*_ROI_/*A*_ROI_). Then, the mean-corrected LacI, histone modification, CENP-A^HCP−3^, NDC-80 or RNA Pol II Ser5^P^ signal was normalized with the mean-corrected DAPI signal in the same ROI (e.g. normalized mean corrected LacI signal *N*_ROI LacI_ = *c*_ROI LacI/_*c*_ROI DAPI_).

### Live-cell imaging

Worms were dissected in M9 buffer droplets to release embryos. Embryos were mounted on agarose pads for imaging. Live images were taken with a Carl Zeiss LSM710 laser scanning confocal microscope with an EC Plan-Neofluar 40× Oil objective and PMT detectors. Stacks with 12 to 14 × 1.8 μm planes were scanned for each embryo in a 3.8× zoom and a 2-min or 30-s interval, or stacks with 25 × 0.6 μm planes were scanned for each embryo in a 1.6× zoom. Red and green channels were scanned simultaneously with a 2.55–6.3 μs pixel dwell time and 31–62 μm pinhole. The images were saved in 16 bits format.

AC segregation scoring was performed as described previously [34]. Every dividing cell that contains at least one AC was counted as one sample. Each division was categorized as either containing at least a segregating AC or containing (all) non-segregating AC(s). Segregating ACs were defined as those that aligned with the metaphase plate and segregated equally with endogenous chromosomes during anaphase. Non-segregating ACs include those that did not align with the metaphase plate, or missegregated during anaphase. The segregation rate was then calculated as the number of dividing cells containing segregating ACs over the total number of dividing cells containing ACs.

### Embryo permeabilization and drug treatment

To obtain permeabilized *C. elegans* embryos, the T01H3.4 clone from the Ahringer library [77] was used for *perm*-*1* RNAi by feeding. Bacteria expressing dsRNA targeting *perm*-*1* were grown overnight at 37 °C in LB with 100 mg/ml ampicillin. The overnight culture was diluted 1:50 in LB with 100 mg/ml ampicillin and grown at 37 °C until the culture reached an OD600 around 0.4 (2.5–3 h). The bacterial culture (200 ml/plate) was spread onto NGM agar plates containing 0.01 mM IPTG. Plates were dried in a sterile hood for 1 h and left at room temperature for 4 additional hours to induce the RNA expression [78]. Around 30 L4 stage worms were then transferred onto the plate and incubated overnight at 20 °C for 12–14 h. Then, a mixture of purified p64xLacO plasmid (100 ng/μl) and pRF4 plasmid (50 ng/μl) was co-injected into the gonad of adult worms as described. Worms were recovered at 20 °C for 6 to 8 h on another *perm*-*1* feeding RNAi plate before subsequent immunofluorescence or live imaging of newly formed ACs. For immunofluorescence, α-amanitin (Abcam ab144512) was diluted to the working concentration (200 μg/ml) in 0.7× Egg salt buffer (1× Egg salt buffer: 118 mM NaCl, 40 mM KCl, 3.4 mM MgCl_2_, 3.4 mM CaCl_2_, 5 mM HEPES pH 7.4). Embryos were dissected in 200 μg/ml α-amanitin and incubated for 30 min before performing immunofluorescence. 2 μl Latex beads (15 μm, 74964 Sigma-Aldrich) were added to protect the permeabilized embryos from damage by the coverslip. Immunofluorescence was performed as described above. For live imaging, the microdevice well was filled with 80 μl 0.7× Egg salt buffer [78]. Worms were dissected on the dissection board, and the embryos were swept to the wells by an eyelash tool. The medium in the well was exchanged by removing the existing medium using a syringe and adding fresh medium with 200 μg/ml α-amanitin with a pipette, and incubated for 30 min. Then, live imaging of the embryos was performed as described above.

## Additional files

**Additional file 1: Fig. 1.** Propagated AC construction and AC transmission rate in progeny. (A) Schematic diagram of the experimental set up to construct first-generation ACs for imaging and propagated ACs by selecting the Roller phenotype after co-injection of a mixture of p64xLacO plasmid and pRF4 plasmid. The transmission rate of Roller progeny was measured in multiple F1 lines in either GFP::LacI- and GFP::LacI::HDA-1-tethering strains. (B) Bar graph showing the percentage of Roller progeny produced by each Roller worm derived from 4 different F1 lines in GFP::LacI- or GFP::LacI::HDA-1-tethering strain. The number of worms (*n*) analyzed in each line was indicated.

**Additional file 2: Fig. 2.** Histone acetylations on propagated ACs and endogenous chromosomes at different cell stages. (A) Immunofluorescence of H3K9ac on propagated ACs and endogenous chromosomes at different cell stages in GFP::LacI-tethering strain. Cropped images containing ACs and endogenous chromosomes (Endo Chr.) were shown. Embryos were stained with antibody against H3K9ac (red), antibody against LacI (green) and DAPI (blue), shown separately and merged. Scale bar represents 1 μm for both ACs and endogenous chromosomes. Quantification of IF signals. Histone modification signals were normalized with DAPI signals, and the average normalized histone modification signal intensity was calculated. The number of cells (*n*) analyzed was indicated. Error bars indicate 95% confidence interval (CI) for the mean. NS means not significant. Therefore, the propagated ACs and endogenous chromosomes at different cell stages were grouped, respectively, in Fig. 1C. (B) Immunofluorescence of H4ac on propagated ACs and endogenous chromosomes at different cell stages in GFP::LacI-tethering strain. Cropped images containing ACs and endogenous chromosomes (Endo Chr.) were shown. Embryos were stained with antibody against H4ac (red), antibody against LacI (green) and DAPI (blue), shown separately and merged. Scale bar represents 1 μm for both ACs and endogenous chromosomes. Quantification of IF signals. Histone modification signals were normalized with DAPI signals, and the average normalized histone modification signal intensity was calculated. The number of cells (*n*) analyzed was indicated. Error bars indicate 95% confidence interval (CI) for the mean. NS means not significant. Therefore, the propagated ACs and endogenous chromosomes at different cell stages were grouped, respectively, in Fig. 1D.

**Additional file 3: Fig. 3.** Newly formed ACs at different cell stages contain comparable level of histone protein H3 and H4 as endogenous chromosomes. (A) Immunofluorescence of histone protein H3 on first-generation ACs at different cell stages and endogenous chromosomes in GFP::LacI-tethering strain. Cropped images containing ACs and endogenous chromosomes (Endo Chr.) were shown. Embryos were stained with antibody against H3 (red), antibody against LacI (green) and DAPI (blue), shown separately and merged. Scale bar represents 1 μm for both ACs and endogenous chromosomes. Since the signal of H3 staining is apparent only in interphase and prometaphase, signals were quantified at these stages. Quantification of IF signals. Histone protein H3 signals were normalized with DAPI signals, and the average normalized H3 signal intensity was calculated. The number of samples (*n*) analyzed was indicated. Error bars indicate 95% confidence interval (CI) for the mean. NS means not significant by *t* test. (B) Immunofluorescence of histone protein H4 on first-generation ACs at different cell stages and endogenous chromosomes in GFP::LacI-tethering strain. Cropped images containing ACs and endogenous chromosomes (Endo Chr.) were shown. Embryos were stained with antibody against H4 (red), antibody against LacI (green) and DAPI (blue), shown separately and merged. Scale bar represents 1 μm for both AC and endogenous chromosome. Quantification of IF signals. Histone protein H4 signals were normalized with DAPI signals, and the average normalized histone modification signal intensity was calculated. The number of samples (*n*) analyzed was indicated. Error bars indicate 95% confidence interval (CI) for the mean. NS means not significant by *t* test.

**Additional file 4: Fig. 4.** Propagated ACs accumulate histone modification H3K9me3. Immunofluorescence of H3K9me3 on first-generation ACs, and ACs that have been propagated for generations and endogenous chromosomes in GFP::LacI-tethering strain. Cropped images containing ACs and endogenous chromosomes (Endo Chr.) are shown. Embryos were stained with antibody against H3K9me3 (red), antibody against LacI (green) and DAPI (blue), shown separately and merged. Scale bar represents 1 μm for both ACs and endogenous chromosomes. Quantification of IF signals on first-generation and propagated ACs. Histone modification signals were normalized with DAPI signals, and the average normalized histone modification signal intensity was calculated. The number of cells (*n*) analyzed was indicated. Error bars indicate 95% confidence interval (CI) for the mean. ***p < 0.001 by Student’s *t* test.

**Additional file 5: Fig. 5.** The effects of GFP::LacI::HDA-1 on ACs is specific to the deacetylase enzymatic activity of HDA-1. (A) Immunofluorescence of H3K9ac on first-generation ACs at different cell stages and endogenous chromosomes in GFP::LacI- and GFP::LacI::HDA-1(H145A) mutant-tethering strains. Cropped images containing ACs and endogenous chromosomes (Endo Chr.) were shown. Embryos were stained with antibody against H3K9ac (red), antibody against LacI (green) and DAPI (blue), shown separately and merged. Scale bar represents 1 μm for both ACs and endogenous chromosomes. Quantification of IF signals. Histone modification signals were normalized with DAPI signals, and the average normalized histone modification signal intensity was calculated. The number of cells (*n*) analyzed was indicated. Error bars indicate 95% confidence interval (CI) for the mean. NS means not significant by *t* test. Black arcs show comparisons between GFP::LacI- and GFP::LacI::HDA-1(H145A) mutant-tethering strain at the same cell stage. The data for GFP::LacI-tethering strain are the same as in Fig. 1C. (B) Immunofluorescence of H4ac on first-generation ACs at different cell stages and endogenous chromosomes in GFP::LacI- and GFP::LacI::HDA-1(H145A) mutant-tethering strains. Cropped images containing ACs and endogenous chromosomes (Endo Chr.) were shown. Embryos were stained with antibody against H4ac (red), antibody against LacI (green) and DAPI (blue), shown separately and merged. Scale bar represents 1 μm for both ACs and endogenous chromosomes. Quantification of IF signals. Histone modification signals were normalized with DAPI signals, and the average normalized histone modification signal intensity was calculated. The number of cells (*n*) analyzed was indicated. Error bars indicate 95% confidence interval (CI) for the mean. NS means not significant. Black arcs show comparisons between GFP::LacI- and GFP::LacI::HDA-1(H145A) mutant-tethering strains at the same cell stage. The data for GFP::LacI-tethering strain are the same as in Fig. 1D.

**Additional file 6: Fig. 6.** Active transcription histone modification maker H3K4me2 is found on newly formed ACs. Immunofluorescence of H3K4me2 on first-generation ACs at different cell stages, and ACs that have been propagated for generations and endogenous chromosomes in GFP::LacI-tethering strain. Cropped images containing ACs and endogenous chromosomes (Endo Chr.) were shown. Embryos were stained with antibody against H3K4me2 (red), antibody against LacI (green) and DAPI (blue), shown separately and merged. Scale bar represents 1 μm for both ACs and endogenous chromosomes. Quantification of IF signals. Histone modification signals were normalized with DAPI signals, and the average normalized histone modification signal intensity was calculated. The number of cells (*n*) analyzed was indicated. Error bars indicate 95% confidence interval (CI) for the mean. ***p < 0.001, **p < 0.01 and *p < 0.05 by Student’s *t* test. NS means not significant. Arcs show comparisons between ACs at different stages.

**Additional file 7: Fig. 7.** RNA polymerase II-mediated transcription affects the histone H3K9 and H4 acetylation level on newly formed ACs in early cell stage. (A) A schematic diagram of the experimental set up to treat permeable embryos with alpha-amanitin, followed by immunofluorescence. (B) Immunofluorescence of H3K9ac on first-generation ACs at different cell stages, and endogenous chromosomes in GFP::LacI-tethering strain without and with alpha-amanitin treatment. Cropped images containing ACs and endogenous chromosomes (Endo Chr.) were shown. Embryos were stained with antibody against H3K9ac (red), antibody against LacI (green) and DAPI (blue), shown separately and merged. Scale bar represents 1 μm for both ACs and endogenous chromosomes. Quantification of IF signals. H3K9ac signals were normalized with DAPI signals, and the average normalized H3K9ac signal intensity was calculated. The number of cells (*n*) analyzed was indicated. Error bars indicate 95% confidence interval (CI) for the mean. ***p < 0.001 by Student’s *t* test. NS means not significant. Black arcs show comparisons between without and with alpha-amanitin treatment at the same cell stage. The data for GFP::LacI-tethering strain without alpha-amanitin treatment are the same as in Fig. 1C. (C) Immunofluorescence of H4ac on first-generation ACs at different cell stages, and endogenous chromosomes in GFP::LacI-tethering strain without and with alpha-amanitin treatment. Cropped images containing ACs and endogenous chromosomes (Endo Chr.) were shown. Embryos were stained with antibody against H4ac (red), antibody against LacI (green) and DAPI (blue), shown separately and merged. Scale bar represents 1 μm for ACs and endogenous chromosomes. Quantification of IF signals. H4ac signals were normalized with DAPI signals, and the average normalized H4ac signal intensity was calculated. The number of cells (*n*) analyzed was indicated. Error bars indicate 95% confidence interval (CI) for the mean. ***p < 0.001, **p < 0.01 and *p < 0.05 by Student’s *t* test. NS means not significant. Black arcs show comparisons between without and with alpha-amanitin treatment at the same cell stage. The data for GFP::LacI-tethering strain without alpha-amanitin treatment are the same as in Fig. 1D.

**Additional file 8.** Worm strains and their genotypes used in this study.
