# Supplementary figures and images for: Histone H3K9 and H4 Acetylations and Transcription Facilitate the Initial CENP-AHCP−3 Deposition and De Novo Centromere Establishment in Caenorhabditis elegans Artificial Chromosomes
Source: Epigenetics Chromatin. 2018 Apr 13;11:16. doi: 10.1186/s13072-018-0185-1 (PMC5898018; doi:10.1186/s13072-018-0185-1)

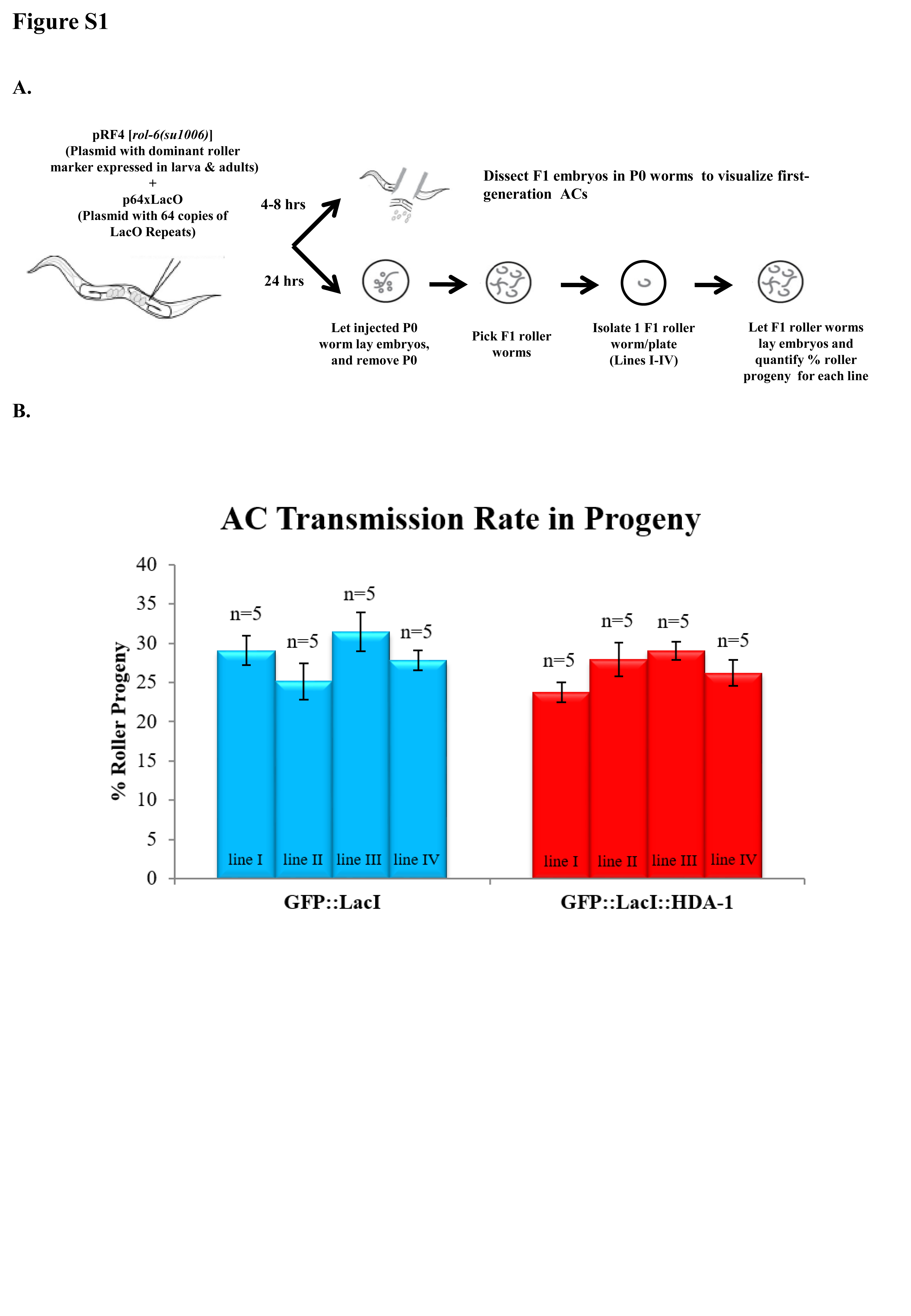

Supplement: Supplementary file 1 — Additional file 1: Fig. 1. Propagated AC construction and AC transmission rate in progeny. (A) Schematic diagram of the experimental set up to construct first-generation ACs for imaging and propagated ACs by selecting the Roller phenotype after co-injection of a mixture of p64xLacO plasmid and pRF4 plasmid. The transmission rate of Roller progeny was measured in multiple F1 lines in either GFP::LacI- and GFP::LacI::HDA-1-tethering strains. (B) Bar graph showing the percentage of Roller progeny produced by each Roller worm derived from 4 different F1 lines in GFP::LacI- or GFP::LacI::HDA-1-tethering strain. The number of worms (n) analyzed in each line was indicated. [file 13072_2018_185_MOESM1_ESM.tif]

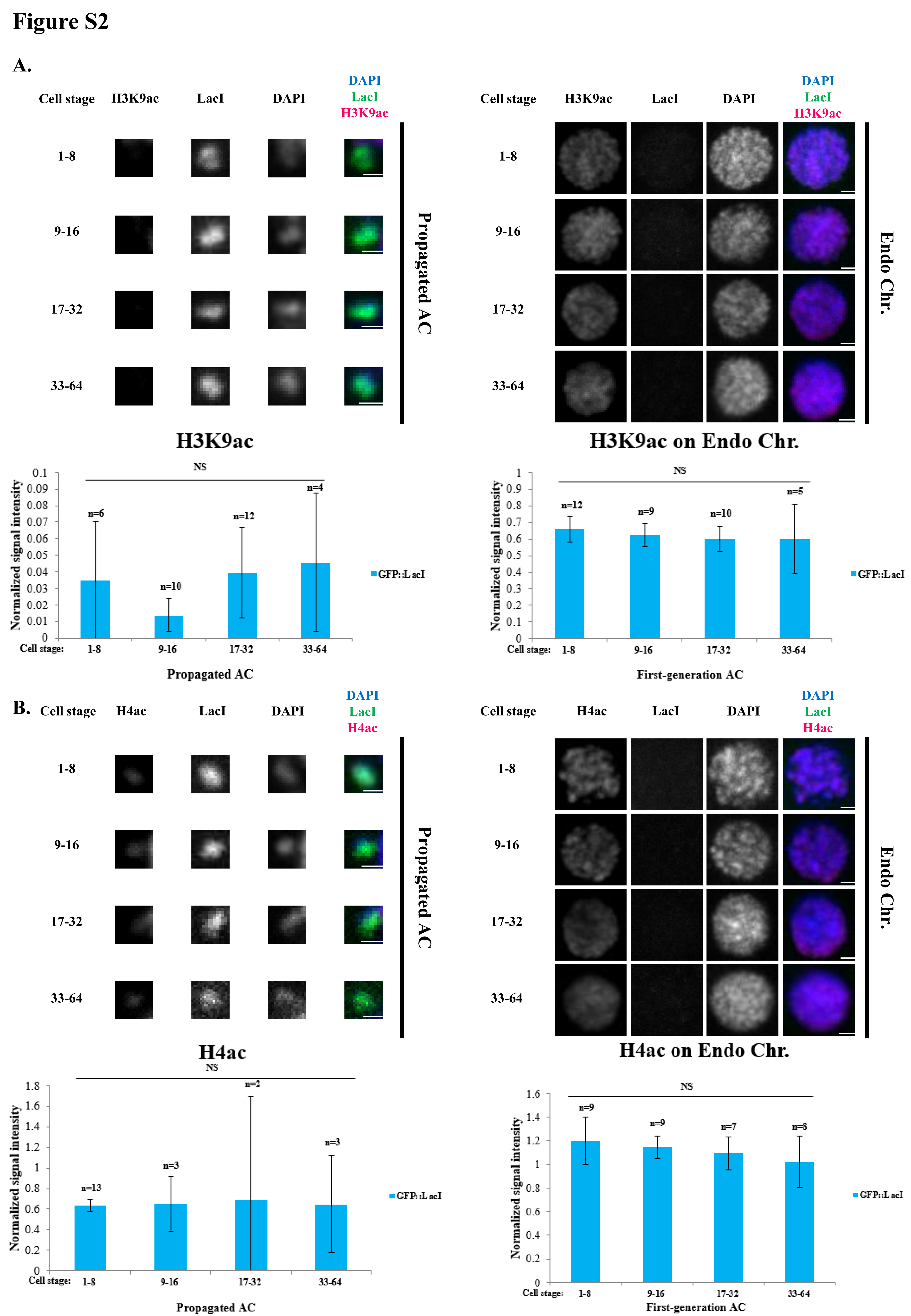

Supplement: Supplementary file 2 — Additional file 2: Fig. 2. Histone acetylations on propagated ACs and endogenous chromosomes at different cell stages. (A) Immunofluorescence of H3K9ac on propagated ACs and endogenous chromosomes at different cell stages in GFP::LacI-tethering strain. Cropped images containing ACs and endogenous chromosomes (Endo Chr.) were shown. Embryos were stained with antibody against H3K9ac (red), antibody against LacI (green) and DAPI (blue), shown separately and merged. Scale bar represents 1 μm for both ACs and endogenous chromosomes. Quantification of IF signals. Histone modification signals were normalized with DAPI signals, and the average normalized histone modification signal intensity was calculated. The number of cells (n) analyzed was indicated. Error bars indicate 95% confidence interval (CI) for the mean. NS means not significant. Therefore, the propagated ACs and endogenous chromosomes at different cell stages were grouped, respectively, in Fig. 1C. (B) Immunofluorescence of H4ac on propagated ACs and endogenous chromosomes at different cell stages in GFP::LacI-tethering strain. Cropped images containing ACs and endogenous chromosomes (Endo Chr.) were shown. Embryos were stained with antibody against H4ac (red), antibody against LacI (green) and DAPI (blue), shown separately and merged. Scale bar represents 1 μm for both ACs and endogenous chromosomes. Quantification of IF signals. Histone modification signals were normalized with DAPI signals, and the average normalized histone modification signal intensity was calculated. The number of cells (n) analyzed was indicated. Error bars indicate 95% confidence interval (CI) for the mean. NS means not significant. Therefore, the propagated ACs and endogenous chromosomes at different cell stages were grouped, respectively, in Fig. 1D. [file 13072_2018_185_MOESM2_ESM.tif]

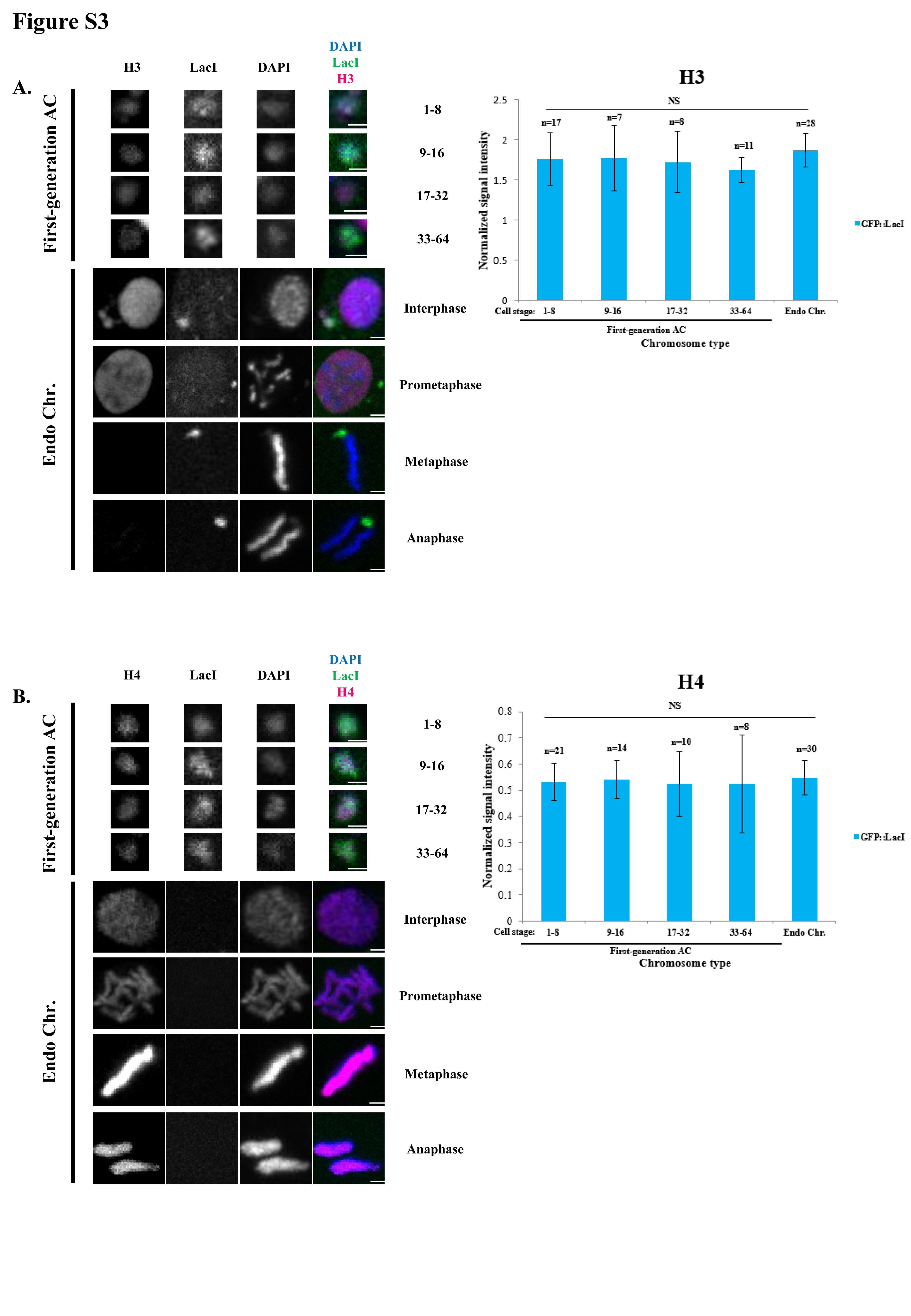

Supplement: Supplementary file 3 — Additional file 3: Fig. 3. Newly formed ACs at different cell stages contain comparable level of histone protein H3 and H4 as endogenous chromosomes. (A) Immunofluorescence of histone protein H3 on first-generation ACs at different cell stages and endogenous chromosomes in GFP::LacI-tethering strain. Cropped images containing ACs and endogenous chromosomes (Endo Chr.) were shown. Embryos were stained with antibody against H3 (red), antibody against LacI (green) and DAPI (blue), shown separately and merged. Scale bar represents 1 μm for both ACs and endogenous chromosomes. Since the signal of H3 staining is apparent only in interphase and prometaphase, signals were quantified at these stages. Quantification of IF signals. Histone protein H3 signals were normalized with DAPI signals, and the average normalized H3 signal intensity was calculated. The number of samples (n) analyzed was indicated. Error bars indicate 95% confidence interval (CI) for the mean. NS means not significant by t test. (B) Immunofluorescence of histone protein H4 on first-generation ACs at different cell stages and endogenous chromosomes in GFP::LacI-tethering strain. Cropped images containing ACs and endogenous chromosomes (Endo Chr.) were shown. Embryos were stained with antibody against H4 (red), antibody against LacI (green) and DAPI (blue), shown separately and merged. Scale bar represents 1 μm for both AC and endogenous chromosome. Quantification of IF signals. Histone protein H4 signals were normalized with DAPI signals, and the average normalized histone modification signal intensity was calculated. The number of samples (n) analyzed was indicated. Error bars indicate 95% confidence interval (CI) for the mean. NS means not significant by t test. [file 13072_2018_185_MOESM3_ESM.tif]

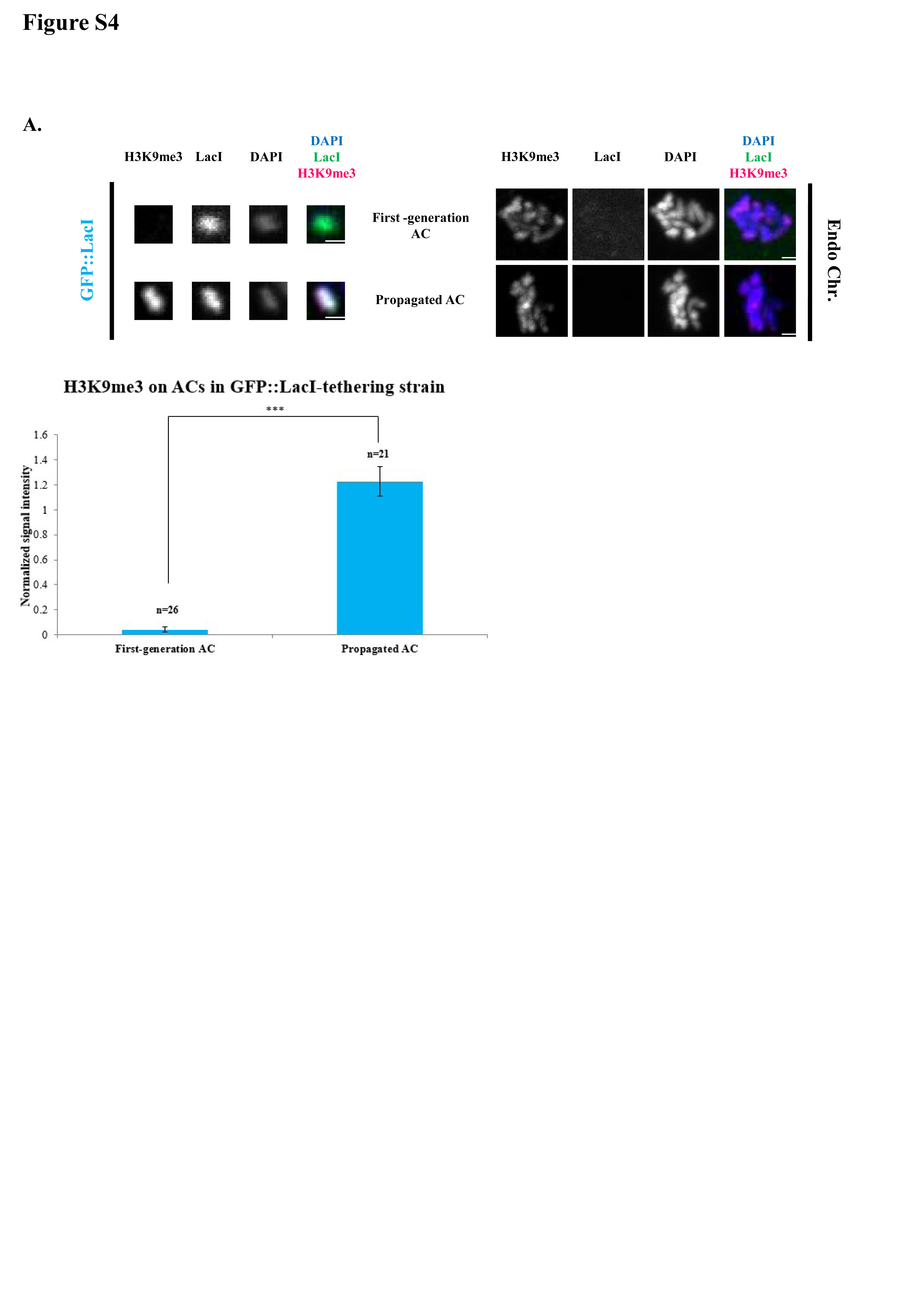

Supplement: Supplementary file 4 — Additional file 4: Fig. 4. Propagated ACs accumulate histone modification H3K9me3. Immunofluorescence of H3K9me3 on first-generation ACs, and ACs that have been propagated for generations and endogenous chromosomes in GFP::LacI-tethering strain. Cropped images containing ACs and endogenous chromosomes (Endo Chr.) are shown. Embryos were stained with antibody against H3K9me3 (red), antibody against LacI (green) and DAPI (blue), shown separately and merged. Scale bar represents 1 μm for both ACs and endogenous chromosomes. Quantification of IF signals on first-generation and propagated ACs. Histone modification signals were normalized with DAPI signals, and the average normalized histone modification signal intensity was calculated. The number of cells (n) analyzed was indicated. Error bars indicate 95% confidence interval (CI) for the mean. ***p < 0.001 by Student’s t test. [file 13072_2018_185_MOESM4_ESM.tif]

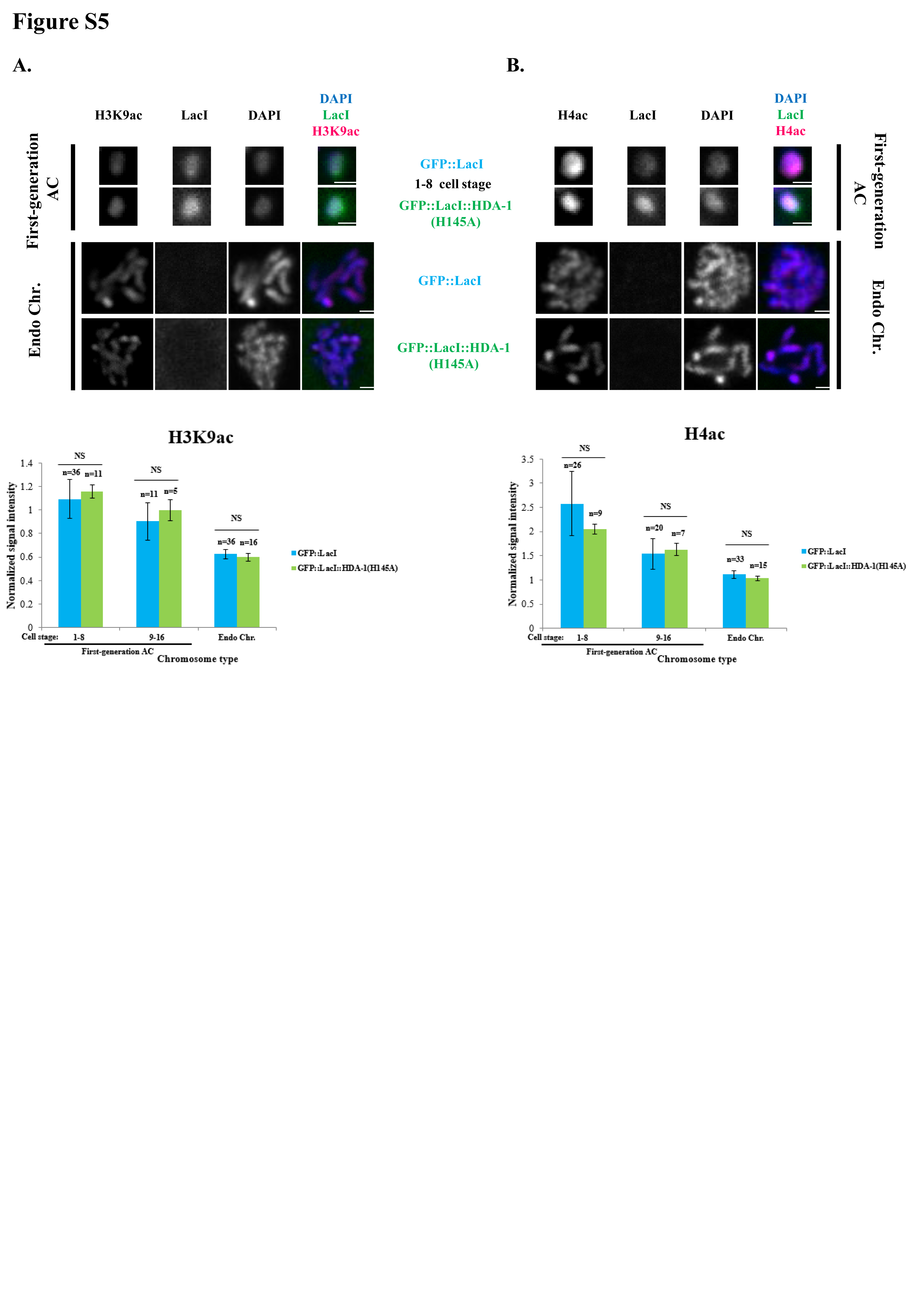

Supplement: Supplementary file 5 — Additional file 5: Fig. 5. The effects of GFP::LacI::HDA-1 on ACs is specific to the deacetylase enzymatic activity of HDA-1. (A) Immunofluorescence of H3K9ac on first-generation ACs at different cell stages and endogenous chromosomes in GFP::LacI- and GFP::LacI::HDA-1(H145A) mutant-tethering strains. Cropped images containing ACs and endogenous chromosomes (Endo Chr.) were shown. Embryos were stained with antibody against H3K9ac (red), antibody against LacI (green) and DAPI (blue), shown separately and merged. Scale bar represents 1 μm for both ACs and endogenous chromosomes. Quantification of IF signals. Histone modification signals were normalized with DAPI signals, and the average normalized histone modification signal intensity was calculated. The number of cells (n) analyzed was indicated. Error bars indicate 95% confidence interval (CI) for the mean. NS means not significant by t test. Black arcs show comparisons between GFP::LacI- and GFP::LacI::HDA-1(H145A) mutant-tethering strain at the same cell stage. The data for GFP::LacI-tethering strain are the same as in Fig. 1C. (B) Immunofluorescence of H4ac on first-generation ACs at different cell stages and endogenous chromosomes in GFP::LacI- and GFP::LacI::HDA-1(H145A) mutant-tethering strains. Cropped images containing ACs and endogenous chromosomes (Endo Chr.) were shown. Embryos were stained with antibody against H4ac (red), antibody against LacI (green) and DAPI (blue), shown separately and merged. Scale bar represents 1 μm for both ACs and endogenous chromosomes. Quantification of IF signals. Histone modification signals were normalized with DAPI signals, and the average normalized histone modification signal intensity was calculated. The number of cells (n) analyzed was indicated. Error bars indicate 95% confidence interval (CI) for the mean. NS means not significant. Black arcs show comparisons between GFP::LacI- and GFP::LacI::HDA-1(H145A) mutant-tethering strains at the same cell stage. The data for [file 13072_2018_185_MOESM5_ESM.tif]

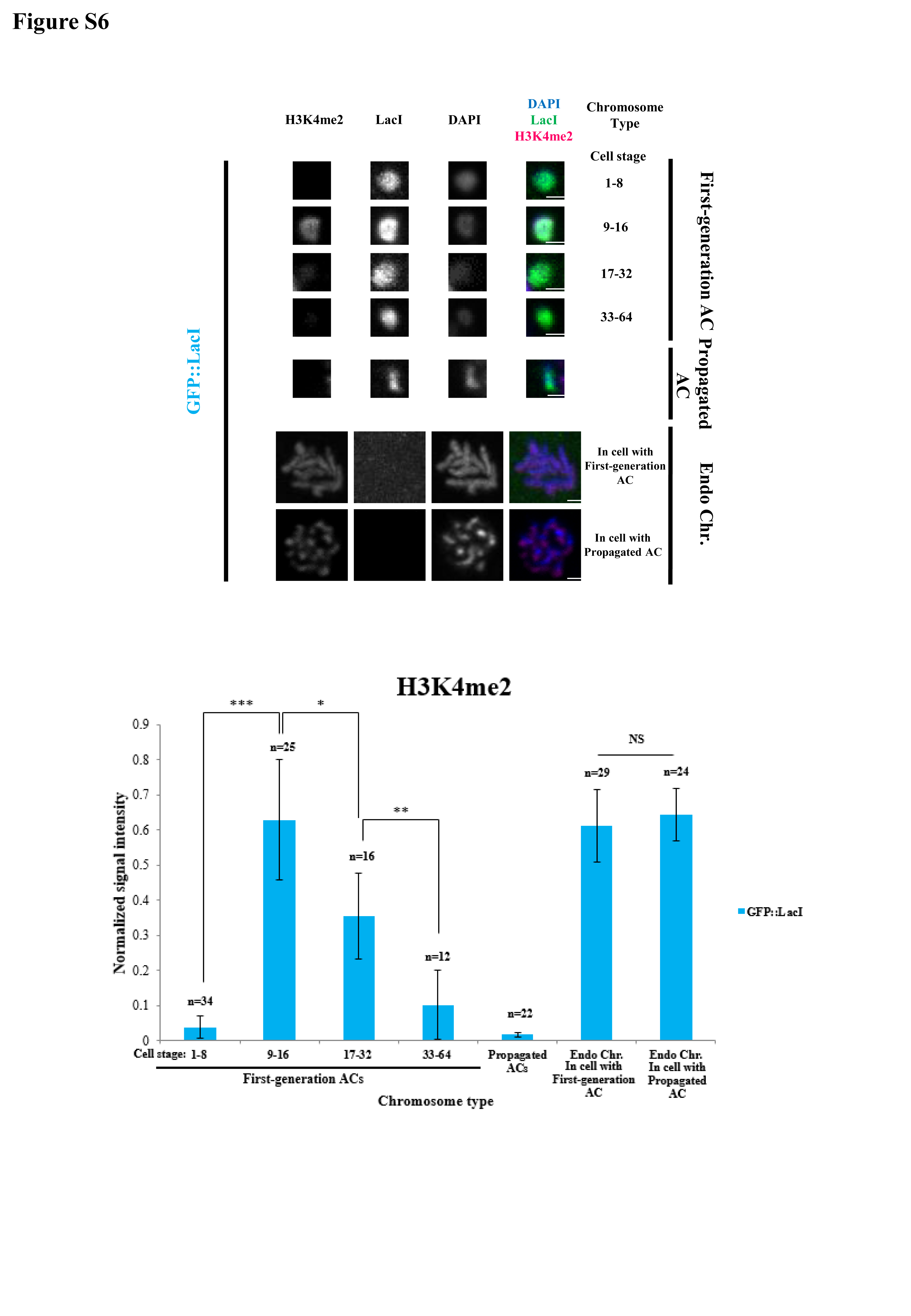

Supplement: Supplementary file 6 — Additional file 6: Fig. 6. Active transcription histone modification maker H3K4me2 is found on newly formed ACs. Immunofluorescence of H3K4me2 on first-generation ACs at different cell stages, and ACs that have been propagated for generations and endogenous chromosomes in GFP::LacI-tethering strain. Cropped images containing ACs and endogenous chromosomes (Endo Chr.) were shown. Embryos were stained with antibody against H3K4me2 (red), antibody against LacI (green) and DAPI (blue), shown separately and merged. Scale bar represents 1 μm for both ACs and endogenous chromosomes. Quantification of IF signals. Histone modification signals were normalized with DAPI signals, and the average normalized histone modification signal intensity was calculated. The number of cells (n) analyzed was indicated. Error bars indicate 95% confidence interval (CI) for the mean. ***p < 0.001, **p < 0.01 and *p < 0.05 by Student’s t test. NS means not significant. Arcs show comparisons between ACs at different stages. [file 13072_2018_185_MOESM6_ESM.tif]

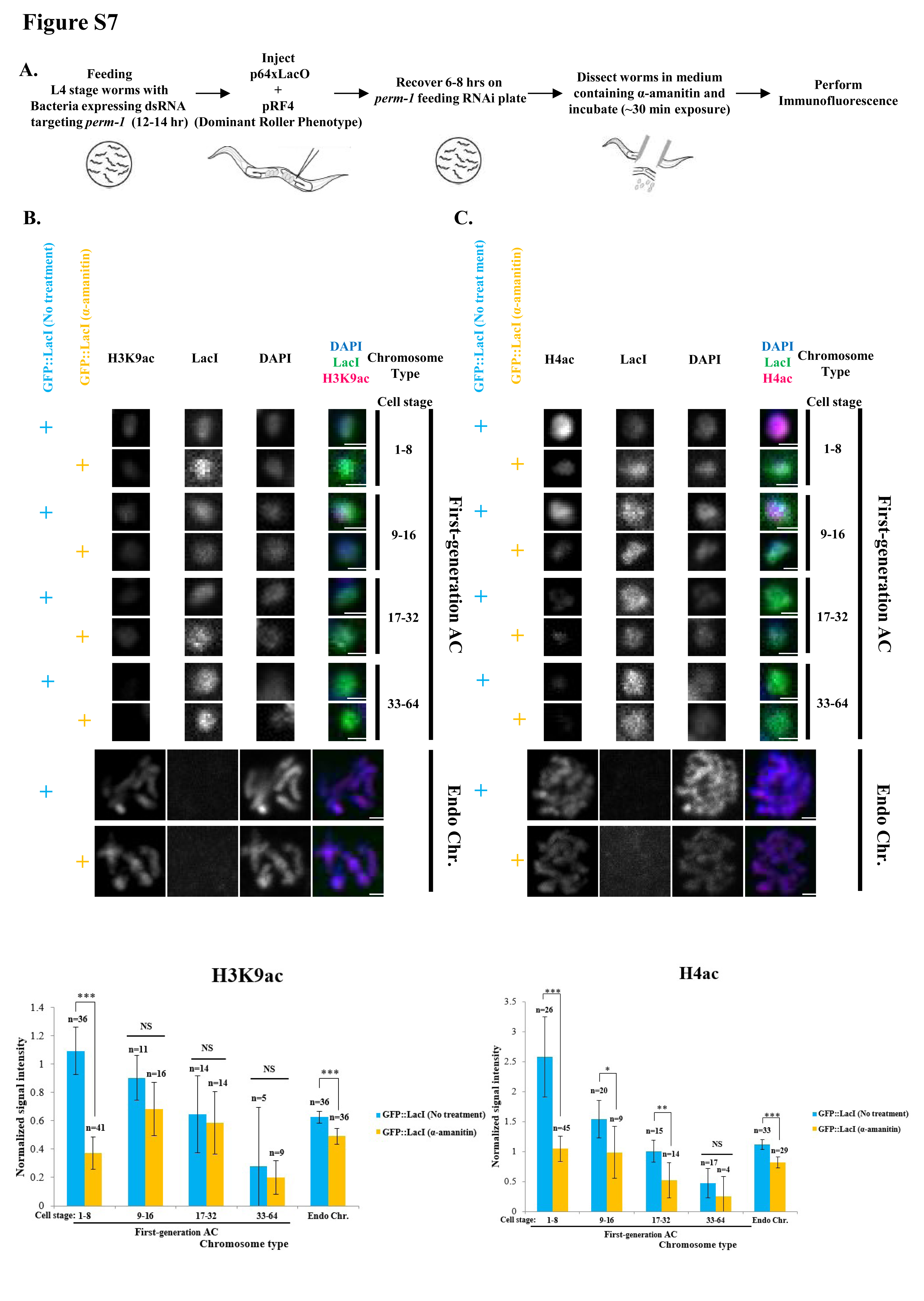

Supplement: Supplementary file 7 — Additional file 7: Fig. 7. RNA polymerase II-mediated transcription affects the histone H3K9 and H4 acetylation level on newly formed ACs in early cell stage. (A) A schematic diagram of the experimental set up to treat permeable embryos with alpha-amanitin, followed by immunofluorescence. (B) Immunofluorescence of H3K9ac on first-generation ACs at different cell stages, and endogenous chromosomes in GFP::LacI-tethering strain without and with alpha-amanitin treatment. Cropped images containing ACs and endogenous chromosomes (Endo Chr.) were shown. Embryos were stained with antibody against H3K9ac (red), antibody against LacI (green) and DAPI (blue), shown separately and merged. Scale bar represents 1 μm for both ACs and endogenous chromosomes. Quantification of IF signals. H3K9ac signals were normalized with DAPI signals, and the average normalized H3K9ac signal intensity was calculated. The number of cells (n) analyzed was indicated. Error bars indicate 95% confidence interval (CI) for the mean. ***p < 0.001 by Student’s t test. NS means not significant. Black arcs show comparisons between without and with alpha-amanitin treatment at the same cell stage. The data for GFP::LacI-tethering strain without alpha-amanitin treatment are the same as in Fig. 1C. (C) Immunofluorescence of H4ac on first-generation ACs at different cell stages, and endogenous chromosomes in GFP::LacI-tethering strain without and with alpha-amanitin treatment. Cropped images containing ACs and endogenous chromosomes (Endo Chr.) were shown. Embryos were stained with antibody against H4ac (red), antibody against LacI (green) and DAPI (blue), shown separately and merged. Scale bar represents 1 μm for ACs and endogenous chromosomes. Quantification of IF signals. H4ac signals were normalized with DAPI signals, and the average normalized H4ac signal intensity was calculated. The number of cells (n) analyzed was indicated. Error bars indicate 95% confidence interval (CI) for the mean. ***p < 0.001, ** [file 13072_2018_185_MOESM7_ESM.tif]
