# Supplementary material for: Histone H3K9 and H4 Acetylations and Transcription Facilitate the Initial CENP-AHCP−3 Deposition and De Novo Centromere Establishment in Caenorhabditis elegans Artificial Chromosomes
Source: Epigenetics Chromatin. 2018 Apr 13;11:16. doi: 10.1186/s13072-018-0185-1 (PMC5898018; doi:10.1186/s13072-018-0185-1)
Supplement: Supplementary file 8 — Additional file 8. Worm strains and their genotypes used in this study. [file 13072_2018_185_MOESM8_ESM.pdf]

Table 1

Strain	Genotype	Reference
OD426	*unc-119(ed3)III; meIs1 [pie-1p::GFP::LacI]; ltIs37 [pie-1p::mCherry::his-58 (pAA64) + unc-119(+)]*	[33]
WYY7	*unc-119(ed3)III; hkuSi2[hda-1p::GFP::LacI::hda-1; cb-unc-119(+)(WYYp33)]II; ltIs37 [pie-1p::mCherry::his-58 (pAA64) + unc-119(+)]*	This study
WYY28	*unc-119(ed3)III; hkuSi6[Phda-1::GFP::LacI::hda-1(H145A)::hda-1 3'UTR; cb-unc-119(+)(WYYp112)]II; ltIs37 [pie-1p::mCherry::his-58 (pAA64) + unc-119(+)]*	This study
